# Women intend to buy, and attend more to, healthy foods in supermarkets: attention to product placement observed in women in a 2-dimensional simulated supermarket environment

**DOI:** 10.1186/s12889-026-27679-5

**Published:** 2026-05-18

**Authors:** Sarah Muir, Sarah Jenner, Sarah Crozier, Charlotte E. Lee, Hayward J. Godwin, Hannah Payne, Olivia M. Maynard, Marcus R. Munafò, Christina Vogel, Janis Baird

**Affiliations:** 1 Medical Research Council Lifecourse Epidemiology Centre, University of Southampton, Southampton General Hospital, Tremona Road, Southampton, SO16 6YD UK; 2 School of Health Sciences, University of Southampton, Highfield Campus, University Road, Southampton, S017 1BJ UK; 3 NIHR Applied Research Collaboration Wessex, Southampton Science Park, Innovation Centre, 2 Venture Road, Chilworth, Southampton, SO16 7NP UK; 4 National Institute for Health Research Southampton Biomedical Research Centre, Southampton Centre for Biomedical Research, Southampton General Hospital, Tremona Road, Southampton, SO16 6YD UK; 5 School of Psychology, University of Southampton, Highfield Campus, University Road, Southampton, S017 1BJ UK; 6 School of Psychological Science, University of Bristol, Beacon House, Queens Road, Bristol, BS8 1QU UK; 7 Vice Chancellor’s Office, The Chancellors’ Building, University of Bath, Claverton Down, Bath, BA2 7AY UK; 8 Centre for Food Policy, City St Georges, University of London, Myddelton Street, London, EC1R 1UW UK

**Keywords:** Food, Food policy, Food retail, 2-dimensional simulated supermarket, Behaviour, Visual attention

## Abstract

**Background:**

Research in real supermarkets can be challenging due to competing health and business agendas. This study used a 2-dimensional (2D) simulated supermarket to investigate whether visual attention to, and intended purchase of, products in prominent store locations (checkout, end-of-aisle, store entrance) differed for healthy, unhealthy or non-food items. The study also explored if these findings were modified by participants’ level of educational attainment.

**Methods:**

Women from Wessex, UK, took part in one of two trials. Phase 1: 201 women completed an online questionnaire in which all participants viewed simulated supermarket journeys depicting healthy, unhealthy and non-food product routes, starting at the store entrance and ending at the checkout, in a random order. Participants clicked on items (i) that they were interested in and then (ii) that they wished to purchase. Phase 2: 71 women participated in an eye-tracking study in which all participants again viewed all journey types, followed by a series of side-by-side preferential looking scenes to allow comparison of attention between the three product types, presented in a random order. Twelve participants took part in semi-structured interviews to better understand their views on what influences product attention; data were analysed using thematic analysis.

**Results:**

Phase 1: Participants demonstrated higher attention to food items compared to non-food items but their intention to purchase was higher for healthy foods than both unhealthy and non-food items. Phase 2: there was no difference in attention across product types during the journey task. In the preferential looking task, participants spent more time viewing healthy products than unhealthy or non-food products. Participants also exhibited higher intention to purchase healthy products than unhealthy or non-food items. Interview participants used shopping lists and avoided certain aisles to prevent unhealthy purchases. They wanted to see healthy foods or essential non-food options at prominent store locations.

**Conclusions:**

Women intend to make more healthy purchases than unhealthy purchases when they shop but supermarket strategies to promote less healthy foods in prominent locations in stores may undermine this intent. These findings support the use of prominent positioning of healthier items in stores as a useful strategy to increase healthy sales and support current and planned food policies.

**Trial registration:**

This was a quasi-experimental study of participants’ attention in an online questionnaire (phase 1) and an eye-tracking study (phase 2) and trial registration was not required. Participants did not receive an intervention.

**Supplementary Information:**

The online version contains supplementary material available at 10.1186/s12889-026-27679-5.

## Introduction

### Background

Obesity and poor diet constitute two of the greatest threats to population health and directly contribute to morbidity and mortality [1, 2]. Two-thirds of adults living in England live with overweight and this number increases to 75% in the most deprived areas [3]. Currently, 9.6% of children aged 4–5 years and 22.1% of children aged 10–11 years living in England live with obesity and the number is twice as high in the most deprived areas compared to the least deprived [4]. In 2021 it was estimated that overweight and obesity cost the country £98 billion with a third of that cost attributed to National Health Service costs and costs related to lower productivity [5].

The diets of many adults and children in the UK are high in fat, sugar and salt (HFSS) or ultra-processed foods [6]. Statistics published in 2025 confirm that only 17% of adults and 9% of adolescents in the UK meet the five portions a day recommendation for fruit and vegetables whilst 80% of adults eat more than the recommended intake of free sugars [7]. Relatedly, those who have a lower level of education are more likely to have a poorer diet than those with a higher education who shop at the same places, likely as a result of financial constraints, less knowledge of food health and lower levels of social support [8]. Manufacturers of ultra-processed foods purposefully add high levels of sugar and salt as well as additives such as flavourings, colourings and emulsifiers to create foods that are appealing and convenient for customers [9]. Driven by a highly competitive market and low-cost ingredients, food companies invest in and develop high numbers of HFSS and ultra-processed foods because consumers prefer foods that are affordable, available and convenient [10] Supermarkets, where over 90% of UK grocery sales occur [11], employ a wide range of strategies designed to manipulate shoppers into increasing the amount that they spend on certain products [12, 13] and in 2023, HFSS products accounted for ~£18 billion of food and drink sales in the UK [14]. Healthier foods (such as fruit and vegetables) are more than double the cost per calorie than HFSS [15]. Furthermore, whilst the cost of less healthy foods increased by 11% between 2022 and 2024, the cost of healthy foods in those same years rose by 21% further highlighting the disparity of costs between the two [15]. It is therefore not surprising that many families’ diets do not meet Government recommendations for health.

Less healthy, HFSS and ultra-processed foods are typically marketed in key store locations (such as store entrances, end-of-aisles or checkouts) as a marketing tactic to increase sales of these items [16, 17]. Scientific evidence indicates that increased exposure to prominent location displays of HFSS products in retail outlets is associated with less healthy choices, increased BMI and dietary inequalities [18, 19]. However, by replacing HFSS products with healthier or non-food items in prominent areas in supermarkets, families can make healthier choices [8, 19–22]. One systematic review highlights how interventions that increase the number of healthy food items in prominent locations led to more sales of healthier foods and better dietary behaviours [19]. Given the widening inequalities in dietary health and obesity rates, some studies have tried to explore the influence of socioeconomic status on intervention effectiveness but with no conclusive results [19, 23].

Regulating the prominent placement of foods in retail environments has also become a policy concern. In 2022, Berkeley, a city in California, mandated that only non-sweetened drinks and foods low in salt and sugar could be placed at checkouts; one year after policy implementation the healthfulness of store checkout areas significantly increased [24]. Similarly, in England, UK government policy recognises that to equitably improve dietary health, interventions need to target the environments where food is purchased rather than rely on individual action [25]. In October 2022, the Government in England banned the placement of HFSS foods and drinks in prominent locations in large retailers [26], an initiative that is also planned in Scotland and Wales in 2026 [27, 28].

Research using adequately powered clustered randomised controlled trials in supermarkets is limited, largely due to the complexity of study design and the large number of stores required to produce generalisable results [29]. Evaluating changes in supermarket layout is notoriously challenging due to differing health and business agendas and randomisation at the store level requires a level of commitment that is problematic in such a highly competitive commercial setting [30]. Consequently, 2D supermarket images are widely used and validated in nutritional and behavioural research to replicate in-store supermarket products and displays [31–35]. For example, a substantial body of work has used 2D images of supermarket products embedded within realistic shelf and supermarket contexts to examine how consumers attend to and interpret nutrition information (e.g., traffic-light labels, warning labels and Health Star Ratings) and how this influences attention, perceived healthfulness, and purchase intentions [e.g., 31–34]. Using image-based simulations of supermarket shelving is cost-effective and allows researchers to isolate visual attention and health-related decision-making without the confounding influence of in-store factors such as lighting, noise, pricing, promotions, or shelf-edge marketing [36, 37].

The current study also employs objective eye-tracking measures, a method that provides a deeper understanding of the cognitive mechanisms underlying health-related behaviours [38]. Such techniques have been used to examine how product placement guides customers’ visual attention for placement on supermarket shelves and how branding and packaging affects attention [39–43]. For example, Bialkova et al. found that customers look more times and longer at products to the right of a shelf in supermarkets [39]. Eye-tracking glasses worn by customers in supermarkets have highlighted that if an end of aisle display captured customer’s attention, then they were more likely to visit the aisle and this effect was larger for HFSS items [43]. Eye-tracking research using 2D images of retail shelves, demonstrates that consumers will look at supermarket products for longer periods of time and more often before they choose a product [44]. Sorensen’s [35] mapping of shopper behaviour demonstrated that visual attention in supermarkets is concentrated in three key “hot zones”: the store entrance, end-of-aisle displays, and the checkout area. His work, based on in-store tracking, photographic documentation, and 2D planograms, showed that shoppers slow down and visually recalibrate in the entrance zone, are highly susceptible to visual interruption and impulse triggers at aisle-ends, and display prolonged, captive attention at the checkout [35]. Our study builds directly on this framework by using controlled 2D images of these same three locations to examine how attention varies not only by store zone but also by product healthfulness. Thus, the current study used a 2D simulated supermarket and eye tracking technology to test differences in visual attention and intended purchase of healthy, unhealthy and non-food products placed in prominent in-store locations. We also included a qualitative component, interviewing twelve participants after they completed the eye-tracking studies to understand, from their perspective, what aspects of the simulated supermarket grabbed their attention and how they felt this linked to real-world supermarket settings. Women of childbearing age were the target population because they remain primarily responsible for domestic food tasks such as shopping and cooking [45], and their nutrition status influences the short- and long-term health of their children [46]. Difference in effects by educational attainment was also assessed as a proxy for potential effects on dietary inequalities.

The research objectives were to:

1. Determine whether there are differences in visual attention given to, and intended purchase of, healthy, unhealthy or non-food products placed in prominent locations in a 2D simulated supermarket among a population sample of women with children aged 18–45 years;
2. Establish whether the level of visual attention given to, and intended purchase of, healthy, unhealthy or non-food products placed in prominent locations in a 2D simulated supermarket are modified according to level of educational attainment.

## Methods

### Study design

The APPROVE study consisted of two phases. Both phases used a randomised within-participants design to display simulated supermarket displays to participants and assess visual attention and their intention to purchase across three types of product (healthy, unhealthy, and non-food items) in three different supermarket locations (entrance, end-of-aisle and checkout). Phase 1 used an online survey design to display the supermarket images and collected data on the number of mouse clicks for each product. Phase 2 used a mixed-methods approach involving eye-tracking technology to collect objective gaze data to assess visual attention. The first twelve participants in phase 2 also consented to participate in a short semi-structured qualitative interview about their experiences of product placement and attention in both the simulated and real-world supermarkets.

### The simulated supermarket

The simulated supermarket was made up of a series of still images of supermarket displays at three different locations: checkout, end-of-aisle, store entrance (see Appendix 1). Images were based on real store layouts identified through photos taken by the research team in supermarkets in and around Southampton, UK and the research team’s prior knowledge of prominent placement displays in supermarkets [22, 47]. A graphic designer was employed to create the images; product labels were designed to mimic the colours and shapes of familiar popular brands without using actual brand names. Food products were chosen based on the NOVA Food Classification System, which divides all foods into four groups: (1) unprocessed or minimally processed foods, (2) processed culinary ingredients, (3) processed foods, and (4) ultra-processed foods [48]. The selection of healthy foods was based primarily on group 1, and the selection of unhealthy foods was based primarily on group 4. All food displays included ambient shelf products to ensure comparability. Healthy displays were matched with equivalent unhealthy alternatives, by ensuring that each image adhered to a particular ‘theme’. For example, an end-of-aisle image showing healthy breakfast foods was matched to an equivalent end-of-aisle image showing unhealthy breakfast foods. Non-food items consisted of items selected in the authors’ previous intervention study which looked at the effect on purchasing behaviour when replacing confectionary at checkouts with non-food items such as painkillers, lip balm, and hand sanitiser [22] and through discussions with the retail partner in the previous study based on their knowledge of what could be feasibly stocked. A full list of products included in the study displays is given in supplementary file 1. Two local women, who matched our participant group (women with children between 18 and 45 years), were consulted on the simulated supermarket images and helped to achieve a realistic design.

### Phase 1

#### Setting

Phase 1 was conducted between February and December 2021 during the COVID-19 pandemic. To comply with social distancing measures set out by the UK government, an online survey method was used. Recruitment was conducted across Wessex (counties of Hampshire, Dorset, Wiltshire in South England, UK), but was largely targeted at women in Southampton, a relatively deprived city ranked the 55th most deprived out of 317 local authorities in the UK. Ethical approval for the study was granted by the University of Southampton Faculty of Medicine Ethics Committee and all participants gave informed consent to participate (ERGO 61757).

#### Participants and recruitment

Women were eligible to participate if they: (i) had one or more children, (ii) were aged between 18 and 45 years and (iii) lived in Wessex. We used four methods of recruitment. First, recruitment posters were shared via the official Sure Start Children’s Centre Southampton Facebook page, through local Facebook parentings sites and via targeted paid adverts on Facebook to five deprived towns and cities in Wessex including Southampton, Portsmouth, Bournemouth, Havant, and Poole. Second, once the COVID-19 lockdown was lifted, fliers were shared through various mother and toddler groups in the more deprived areas of Southampton to aim for a sample of the target population; those with low levels of educational attainment. Third, existing relationships with Southampton City Council were also utilised: study adverts were shared with parents via schools by council managers within the more deprived areas of Southampton. Finally, snowball sampling was used: after study participation, women were invited to share details of the study with other women they knew who would be eligible to participate. Women could contact the research team using Facebook messenger, email, or text/call to a dedicated study phone.

#### Procedure

Demographic details were collected from each participant and this information was stored in a Microsoft Access database which was also used to allocate each woman with a unique ID number. Once a woman had been allocated an ID number, she was provided with an information sheet, her ID number and a link to the survey, via email.

The survey was developed by the researchers and hosted on Qualtrics and consisted of a consent form, demographics form, and two stages of simulated supermarket images (supplementary file 2). Images were presented as journeys through a typical supermarket starting with a store entrance, three end-of-aisle images and a checkout image. Participants viewed three variations of these journeys displaying either healthy food, unhealthy food or non-food items. The order in which each product condition was presented was randomised and balanced by Qualtrics to minimise order effects. Randomisation details were blinded to all researchers. The outcome measure in stage 1 was participant *attention*. Participants viewed all three journeys and were asked to “*click on any products that catch your attention*”. Each image was presented for only 10 seconds to ensure that participants acted on their immediate responses. Upon clicking on a product, a blue dot appeared in the exact location of the click, with a maximum of 10 clicks being allowed on each image. In between each image, a generic image showing a blurred view down a supermarket aisle was presented for two seconds to mimic a real store journey. Stage two presented the same three journeys, but the outcome measure was *intention to purchase.* In this stage, participants were instructed to “*click on any products that you would purchase if this was a shopping trip.”* In both stages, outcome was measured by number of clicks on each type of product: healthy; unhealthy; and non-food.

Following completion of the survey, each participant was emailed a £10 Amazon voucher to thank them for their contribution.

#### Statistical analysis

Data were analysed using Stata. Means (SDs) are presented for continuous normally distributed variables and medians (IQRs) for continuous non-normally distributed variables. Categorical variables are summarised using numbers and percentages.

Since there were differing numbers of items at each of the locations used in the journeys (Appendix 1), analyses were performed, and are presented, as number of clicks per 10 items. Differences in numbers of clicks per 10 items between two conditions (e.g., healthy – unhealthy) are compared to zero using one-sample t-tests for normally distributed differences. Wilcoxon sign-rank tests were used for non-normally distributed differences which were seen for clicks on images at the entrance where there were low numbers of products. To explore the effect of educational attainment on the differences in number of clicks, linear regression models were fitted with differences in number of clicks per 10 items as the outcome and educational level as a predictor.

### Phase 2

#### Setting

The study was conducted between October 2022 and March 2023. This was timely because in October 2022 the UK Government had just implemented regulations that banned the placement of HFSS products in all the prominent positions in supermarkets including the areas of interest in this study: store entrance, end-of-aisles and checkouts [26]. The study was conducted at the eye-tracking laboratories at The University of Southampton. Data collection lasted around one hour, and childcare was provided, via an external childcare service if needed so this wasn’t a barrier for the women to participate. Ethical approval was granted by the University of Southampton Faculty of Medicine Ethics Committee (ERGO 76690).

#### Participants and recruitment

The same recruitment methods used in phase 1 were also used in phase 2, including advertising the study via Facebook, local toddler groups and schools in the more deprived areas of Southampton and inviting participants to share study information with other eligible women. Fourteen participants who had previously participated in phase 1 also took part in phase 2 after seeing details of phase 2 in a study dissemination newsletter.

#### Procedure

Study adverts invited potential participants to contact the researcher via email, phone or directly via Facebook. All interested participants were sent an information sheet and encouraged to contact the researcher to arrange a suitable time to attend the eye-tracking laboratories if they were interested. On arrival at the laboratories, participants were given a chance to re-read the study information and ask questions before they signed a consent form.

The study visit was broken into several components: a journey task, a preferential looking task, a post-study questionnaire, and an optional interview until a sample of twelve interview participants was reached.

The journey and preferential looking tasks involved tracking the eye movement behaviour of participants as they viewed the simulated supermarket images. Both of these tasks began with calibration to check that the eye tracker could accurately record the fixations of their eye movement.

The journey task was a replication of the journeys displayed during phase 1, with the addition of eye-tracking. As with phase 1, participants first viewed the three journeys (one each of healthy, unhealthy and non-food) to measure attention. However, unlike phase 1, participants only needed to view the items on the screen in a free-viewing task and were instructed to pay attention to the images on the screen (as opposed to clicking on items in the questionnaire method used in phase 1). Participants were given 10 s to view each image. Viewing time in seconds was used to determine level of visual attention to the predefined regions of the screen that showed different products (rather than the supermarket background). They then revisited the journeys and were asked to click with a computer mouse on any items they saw on screen that they would intend to purchase; they were only able to click once on each product.

Preferential looking tasks are often used in cognitive psychological research to examine intrinsic biases towards stimuli that capture attention. For example, in infants, preferential looking tasks have shown that infants prefer to look at images of human faces rather than of other types [49]. In the context of our current study, participants were presented with randomised images of displays depicting the same location, but different conditions, side-by-side on the computer display. For example, this could include an end-of-aisle display of non-food items on the left of the display alongside an end-of-aisle display of healthy items on the right side of the display (see Appendix 2). The aim was to determine whether different types of items (healthy foods, non-food or unhealthy foods) attract participants’ gaze more than others. Visual attention was again measured by length of time spent viewing each type of image (in seconds). Participants were shown a total of 90 preferential looking images and each image was shown for 10 s. Images were systematically counterbalanced so that participants saw one of six sets of images to control for any positional and pairing effects and ensure that each image appeared equally on each side of the screen and in each pairing across the full sample. During this task, participants were asked to try and remember what they were shown on the screen in preparation for a memory test. This is standard approach used in scene perception studies to ensure meaningful data are collected [50]. Despite these instructions, participants were not subsequently asked to complete a memory test, and it was explained afterwards to participants that attention studies often suggest a memory test will occur to ensure participants adequately engage with the images rather than staring blankly at the screen.

After the two tasks, all participants completed a short demographic questionnaire asking for their age, ethnicity and highest level of education to measure education attainment (see supplementary file 3). All participants received a £25 Amazon or Love2shop voucher to thank them for their participation.

Early participants were invited to take part in an optional short semi-structured interview until we reached our target sample size of twelve participants (i.e. when thematic saturation is achieved). Interviews were audio-recorded and transcribed. Questions focused on what aspects of the products shown in the study they remembered and how far the simulated supermarket matched their real-life supermarket experiences. Interview data were analysed using thematic analysis [51]. A further £10 voucher was given to participants who participated in this part of the study.

#### Statistical analysis

Since there were differing numbers of items across the images (see Appendix 1), analyses of intention were performed, and are presented, as number of clicks per 10 items. Differences in viewing time or numbers of clicks per 10 items between two states (e.g., healthy – unhealthy) are compared to zero using one-sample t-tests for normally distributed differences. Wilcoxon sign-rank tests were used for non-normally distributed differences which were seen for clicks on images at the entrance where there were low numbers of products. To explore the effect of educational attainment on the differences in viewing time or numbers of clicks, linear regression models were fitted with differences in viewing time or number of clicks per 10 items as the outcomes and educational level as a predictor.

In the preferential looking study every participant looked at the same pairs of comparison several times. Therefore, multilevel models were fitted to look at the difference between the viewing time of two states (e.g., healthy – unhealthy), clustered within participant identifier. Again, data were analysed using Stata [52].

## Results

### Descriptive statistics

A total of 207 women with children were recruited to phase 1 between February 2021 and December 2021. Two women made less than 50% progress through the study and four others did not click any images, leaving 201 women in the study sample (Table 1). Women’s median (IQR) age was 36.0 (32.0, 40.0) years. Over half (64.3%) were educated to Higher National Diploma (HND – a vocational qualification equivalent to two years of university study) or degree level. Most (42.8%) of the sample had preschool-aged children. The neighbourhood indices of multiple deprivation identified from women’s postcode were comparable with national levels with 52.5% of women in the lowest half of area deprivation.

**Table 1:** Participant characteristics

	Phase 1		Phase 2	
	*n* = 201	(%)	*N* = 68	(%)
Mean Age in years, (median; IQR)	36.0	(32.0, 40.0)	37.1	(33.3, 41.4)
Educational level, n (%)				
None	3	(1.5%)	0	(0%)
GSCE D or below	1	(0.5%)	0	(0%)
GSCE A*-C	22	(10.6%)	4	(5.9%)
A-Levels	46	(23.1%)	10	(14.7%)
Higher National Diploma	12	(6.0%)	4	(5.9%)
Degree	116	(58.3%)	50	(73.5%)
Age of youngest child, n (%)				
Under 5	86	(42.8%)	34	(50.0%%)
Primary School (5–11 years old)	49	(24.4%)	18	(26.5%)
Secondary school (11–16 years old)	14	(7.0%)	1	(1.5%)
Not reported	52	(25.8%)	15	(22.0%)
White ethnicity, n (%)			54	(79.4%)
Employed, n (%)			55	(82.1%)
Two adults in household, n (%)			56	(82.4%)
Two or more children in household, n (%)			43	(63.2%)

A total of 71 women with children were recruited to phase 2 between October 2022 and March 2023. Of these, 68 women provided demographic data (Table 1). Women’s median (IQR) age was 37.1 (33.3, 41.4) years. Half of the sample had children under the age of five years. Most (72.7%) were educated to HND or degree level. The women were predominantly of white ethnicity (78.8%) and 21.2% received Universal Credit.

### Objective 1: Differences in attention given to, and intention to purchase, healthy, unhealthy or non-food items in prominent locations

#### Phase 1

For participants in phase 1, *attention*, determined by the number of clicks on products participants felt captured their attention, was somewhat higher for both healthy and unhealthy food items than non-food items, mainly driven by differences at checkouts. Table 2 indicates that women clicked on more (mean 0.2 (SD 1.1)) healthy food items than non-food items and more (mean 0.3, SD 0.9) unhealthy foods than non-food items per 10 items in total. Women tended to click on somewhat more unhealthy products than healthy products (mean 0.1, SD 1.1).

**Table 2:** Differences in attention and intention by product type and location (*n* = 201)

	Healthy – unhealthy			Non-food - healthy			Non-food - unhealthy		
	Mean (SD)	t (df) or z	*P*	Mean (SD)	t (df) or z	*P*	Mean (SD)	t (df) or z	*P*
Products woman is interested in (attention), number of clicks per 10 items									
Entrance	0 (-2.5, 2.5)*	-2.4	0.02	0 (-2.5, 2.5)*	1.2	0.23	0 (-2.5, 2.5)*	-1.1	0.29
End-of-aisle	0.0 (1.1)	-0.6 (200)	0.56	0.0 (1.2)	-0.1 (200)	0.91	-0.1 (1.0)	-0.8 (200)	0.45
Checkout	-0.1 (1.9)	-0.5 (200)	0.60	-0.8 (1.8)	-6.7 (200)	< 0.0001	-0.9 (1.6)	-7.8 (200)	< 0.0001
Total	-0.1 (1.1)	-1.1 (200)	0.28	-0.2 (1.1)	-2.8 (200)	0.005	-0.3 (0.9)	-4.6 (200)	< 0.0001
*Products woman would like to buy (intention)*,* number of clicks per 10 items*									
Entrance	0 (-2.5, 2.5)*	2.7	0.008	0 (0, 2.5)*	2.9	0.003	2.5 (0, 2.5)*	4.7	< 0.0001
End-of-aisle	0.5 (1.3)	5.0 (200)	< 0.0001	-0.4 (1.2)	-4.2 (200)	< 0.0001	0.1 (1.1)	1.3 (200)	0.20
Checkout	0.1 (2.3)	0.3 (200)	0.73	-0.5 (1.8)	-3.8 (200)	0.0002	-0.4 (1.8)	-3.5 (200)	0.0007
Total	0.4 (1.4)	4.0 (200)	0.0001	-0.4 (1.1)	-5.2 (200)	< 0.0001	0.0 (1.1)	-0.1 (200)	0.92

*Medians and IQRs (not appropriate to calculate means and SDs due to low number of clicks at entrance (due to low numbers of products))

*Intention to purchase*, measured by clicks on products they would purchase if this was a shopping trip, was higher for healthy foods than both unhealthy foods and non-food items, mainly driven by differences at end-of-aisles, and higher for non-food than unhealthy foods at store entrances. Women clicked on more (mean 0.4, SD 1.4) healthy food items than unhealthy items and more (mean 0.4, SD 1.1) healthy foods than non-food items per 10 items in total (Table 2). There were no clear differences between unhealthy and non-food products except at store entrances where women clicked on more (median 2.5, IQR 0, 2.5) non-food items than unhealthy.

### Phase 2

#### Journey task

The journey task asked participants to simply view the supermarket images depicting a typical supermarket journey (to measure visual attention) and then view them again whilst clicking on items they would choose to buy (to measure intention to purchase). In contrast to phase 1, there were no clear differences in *attention* to healthy, unhealthy or non-food items as measured by viewing time in number of seconds. However, as Table 3 indicates, similar to phase 1, *intention to purchase* was higher for healthy food items than unhealthy (mean 0.7, SD 0.9) and non-food (mean 0.7, SD 0.8) per 10 items in total. Specifically, women intended to buy significantly more healthy products than unhealthy products at store entrances (median 2.5, IQR 0, 2.5) and end-of-aisles (mean 0.6, SD 0.9) and more healthy products than non-food products at end-of-aisles (mean − 0.8, SD 0.8).

**Table 3:** Differences in attention and intention by product type and location (*n* = 71)

	Healthy – unhealthy			Non-food - healthy			Non-food - unhealthy		
	Mean (SD)	t (df) or z	*P*	Mean (SD)	t (df) or z	*P*	Mean (SD)	t (df) or z	*P*
Viewing time (attention) (seconds)									
Entrance	-0.06 (0.81)	-0.6 (70)	0.54	0.01 (0.18)	0.1 (70)	0.89	-0.04 (0.78)	-0.5 (70)	0.63
End-of-aisle	0.19 (2.01)	0.8 (70)	0.43	0.08 (1.61)	0.4 (70)	0.69	0.27 (1.70)	1.3 (70)	0.19
Checkout	0.10 (1.07)	0.8 (70)	0.41	0.05 (0.79)	0.5 (70)	0.60	0.15 (0.94)	1.4 (70)	0.17
Total	0.24 (2.27)	0.7 (70)	0.46	0.14 (2.24)	0.5 (70)	0.60	0.37 (2.11)	1.5 (70)	0.14
*Products woman would like to buy (intention)*,* number of clicks per 10 items*									
Entrance	2.5 (0, 2.5)*	5.0	< 0.001	0 (-2.5, 0)*	0.1	0.10	0 (0, 2.5)*	3.9	< 0.001
End-of-aisle	0.6 (0.9)	5.7 (70)	< 0.001	-0.8 (0.8)	-7.8 (70)	< 0.001	-0.2 (0.7)	-2.2 (70)	0.03
Checkout	0.6 (1.7)	3.1 (70)	0.003	-0.3 (1.6)	-1.7 (70)	0.08	0.3 (1.1)	2.3 (70)	0.03
Total	0.7 (0.9)	6.4 (70)	< 0.001	-0.7 (0.8)	-6.5 (70)	< 0.001	0.0 (0.7)	0.5 (70)	0.61

*Medians and IQRs (not appropriate to calculate means and SDs due to low number of clicks at entrance (due to low numbers of products)

#### Preferential looking task

Due to technical issues with the eye-tracking software, preferential looking task data were only available for 68 of the 71 women. The preferential looking task presented opposing images next to each other to allow objective comparison between product type as it required participants’ attention to differentiate between two different images. Table 4 indicates higher levels of attention for healthy products over unhealthy and non-food items. Women viewed healthy items for 0.10 (95% CI 0.02, 0.18) seconds more than unhealthy items, and 0.15 (95% CI 0.07, 0.23) seconds more than non-food items, particularly at store entrances. Furthermore, women tended to look at non-food items for 0.26 (95% CI 0.08, 0.43) seconds more than unhealthy items at store checkouts.

**Table 4:** Differences in attention by product type and location (*n* = 68)

	Healthy – unhealthy			Non-food - healthy			Non-food - unhealthy		
	Beta	(95% CI)	*P*	Beta	(95% CI)	*P*	Beta	(95% CI)	*P*
Viewing time (attention) (seconds)									
Entrance	0.43	(0.28, 0.59)	< 0.001	-0.52	(-0.66, -0.37)	< 0.001	-0.08	(-0.23, 0.07)	0.29
End-of-aisle	-0.04	(-0.15, 0.07)	0.43	-0.09	(-0.20, 0.02)	0.10	-0.13	(-0.24, -0.03)	0.01
Checkout	0.20	(0.03, 0.38)	0.02	0.05	(-0.12, 0.23)	0.56	0.26	(0.08, 0.43)	0.004
Total	0.10	(0.02, 0.18)	0.01	-0.15	(-0.23, -0.07)	< 0.001	-0.05	(-0.12, 0.03)	0.26

### Objective 2: To establish whether attention given to, and intention to purchase, healthy, unhealthy or non-food products in prominent supermarket locations are modified according to level of educational attainment

For both phases, the level of educational attainment did not have strong effects on the attention given to products. Furthermore, there were differences in the direction of the effect. For example, in phase 1, for every increase in educational level, women were interested in 0.07 (95% CI -0.07, 0.20) more healthy items than unhealthy items per 10 items. Conversely, in phase 2, for every increase in educational level, women spent slightly more time viewing unhealthy than healthy items (0.05 (95% CI -0.56, 0.47) seconds) (Table 5).

**Table 5:** Education as a predictor of differences between attention and intention to purchase products

Effect of education on differences in clicks and viewing time per 10 items	β	(95% CI)	*P*-value	*n*
PHASE 1 Products woman is interested in (attention), number of clicks per 10 items				
Healthy-unhealthy	0.07	(-0.07, 0.20)	0.27	199
Non-food-healthy	-0.13	(-0.25, -0.01)	0.04	199
Non-food-unhealthy	-0.06	(-0.16, 0.05)	0.30	199
*Products woman would like to buy (intention)*,* number of clicks per 10 items*				
Healthy-unhealthy	0.10	(-0.05, 0.26)	0.20	199
Non-food-healthy	-0.10	(-0.22, 0.03)	0.21	199
Non-food-unhealthy	0.00	(-0.12, 0.13)	0.95	199
PHASE 2 *Viewing time (attention) (seconds)*				
Healthy-unhealthy	-0.05	(-0.56, 0.47)	0.85	68
Non-food-healthy	-0.16	(-0.56, 0.24)	0.43	68
Non-food-unhealthy	-0.21	(-0.62, 0.21)	0.32	68
*Products woman would like to buy (intention)*,* number of clicks per 10 items*				
Healthy-unhealthy	0.09	(-0.09, 0.28)	0.33	68
Non-food-healthy	-0.17	(-0.34, 0.00)	0.04	68
Non-food-unhealthy	-0.08	(-0.21, 0.05)	0.23	68

There were also no strong effects of educational attainment on intention to purchase although for both phases there were small effects to indicate that as women’s education level increased so did women’s intention to purchase healthy products. For phase 1, women intended to purchase 0.10 (95% CI -0.05, 0.26) more healthy items than unhealthy items per 10 items and in phase 2 women intended to purchase 0.09 (95% CI -0.09, 0.28) more healthy than unhealthy items per 10 items.

### Qualitative results

Twelve women took part in a short qualitative interview immediately following their participation in the eye-tracking study. The interviews lasted between 8.42 and 15.55 min. The results highlighted two core themes that aid interpretation of the results from phase 2.

#### Theme 1: Women would prefer more promotion of healthy products

Women reported using strategies to avoid making unhealthy impulse purchases including the avoidance of end-of-aisles where less healthy products would likely be promoted. Participants also described using a shopping list to avoid spending more money than planned or to ensure products purchased fitted with planned meals or diet. Participants expressed a preference for healthier options to be on promotion or in prominent locations especially at store checkouts. Some participants would also like more everyday essentials in prominent locations, particularly items that are likely to be forgotten as they journey around the store such as pain killers.

“At the end-of-aisle stuff, particularly at [supermarket] because it’s all like “Oh, it’s only a pound” then they have that big Clubcard branding you’re lured in a bit more but obviously with the cost of everything going up you have to be this is my list, I need to stick to my list and so not deviate from the list.” (P002).

“I like it when you get to the till and you’re going to pay and sometimes they’ve put the ibuprofen and the paracetamol there because they’re things I always forget” (P304).

#### Theme 2: Factors influencing attention: colour, space, familiarity and predictability

Women generally believed that colour was one of the most memorable features of the products they remembered, especially packaging and brands that incorporated the colours red, pink, orange and yellow. Other noticeable visual aspects were limited displays where it was easier to remember products displayed in the images of the store entrances as these only showed four products (compared to an end–of-aisle image that showed eleven). Products that had larger branding and names were also easier to remember. Another common reason that women gave for why they remembered some products over others was their familiarity with the item or because it was something they would normally buy themselves. However, if a product was in an unexpected space that they were not familiar with (e.g., milk was unexpectedly on an end-of-aisle instead of a chiller) this would also stand out in their memory. Furthermore, the women liked product placement to stay stable and found it “frustrating” or “annoying” if products moved. Related to this, they showed preference for similar items to be grouped together.

“I found that I was drawn to like the brighter colours […] like the eggs, the happy egg company, they’re quite like a bright packet so you would remember it and then you remember the advert with all the chickens running around in the garden like whereas if it had been a plain [supermarket] egg box I probably wouldn’t have remembered it quite so much.” (P002).

“I think the screens that had less products on, I think it was fizzy drinks and toilet paper and that kind of thing, those were a lot easier to remember than the shelves with loads of different products on.” (P307).

“so there’s a few where I automatically looked only at the things I normally buy right my attention was drawn to” (P345).

“the one with like the flax seeds at the top, the eggs and the milk, I was a bit like is that long life milk? Because that’s a really random place to have milk.” (P002).

“That’s something that for me when I go shopping is quite frustrating because I like to go in, I know where my products are, I know what I want to buy, and when they start moving them all around, I know they do it to keep you in the shop longer to buy things but for me, it makes me leave, [laughs] […].” (P310).

## Discussion

This simulated supermarket study showed that women displayed a clear intention to purchase healthy products over unhealthy and non-food products when products were placed in prominent supermarket locations. Women’s’ attention to different product types was mixed with greater attention to healthy products in two of the three experimental measures used. More specifically, the first questionnaire-based study required participants to click on the items that captured their attention whilst shown a series of images to recreate a supermarket journey. This first measure demonstrated higher levels of attention for healthy and unhealthy foods over non-food items, although the size of effect was small. In the second journey task experiment, a replication of phase 1 using eye-tracking technology to track visual attention during a free-viewing task of the images was used. Analysis of visual attention using viewing time in seconds demonstrated no differences in attention between the three conditions. The third experimental measure, asked participants to memorise products shown whilst viewing two simultaneously presented displays. This preferential looking method forces participants’ gaze to differentiate between two product types being displayed in prominent locations at the same time. Results demonstrated higher levels of attention to healthy products over unhealthy and non-food items.

Overall, the quantitative findings indicated a stronger intention to buy healthy products than unhealthy or non-food products. This finding was supported by the qualitative results which highlighted women’s preference for healthier products to be placed at prominent locations. Our research provides both quantitative and qualitative evidence which aligns with previous qualitative insights demonstrating that customers want easier access to healthier foods [53, 54]. Interview participants in a study by Dhuria et al. expressed wanting to make healthier shopping choices; they would often use shopping lists and meal plans with the aim of making healthy purchases but aspects of the supermarket environment including prominent placement and promotions prompted unplanned, less healthy, impulse purchases [54]. Consumers also recognise that pester power from children asking for items can also prompt unintended purchases as a direct result of prominent positioning despite good intentions to buy healthy [54, 55]. Our mixed results on customer’s attention to shopping displays also suggest that the common placement of unhealthy displays undermines their intent to purchase healthy products. Retailers may feel pressure to promote HFSS products in prominent locations due to their perceptions of customer demand and contractual agreements held with suppliers. Retailers may also be concerned that replacing high profit sugary and salty foods with healthier alternatives will be less profitable [56]. Our study suggests that customer attention to prominent displays is similar across product types so there may be little commercial risk to removing unhealthy products from these high footfall areas. This is reinforced by supermarket trials that demonstrate increased purchases of healthy products when these are placed in prominent locations such as the front of store or checkout area [21, 24]. Previous eye-tracking studies have shown that if customer’s shop with a goal to purchase healthier products then equal attention is given to different brands, although attention may be stronger for those with colourful front of pack nutrition labelling [39].

Customers are supportive of Government intervention to change the food system to help them make healthier purchases [53, 55]. Since October 2022, food (placement and promotion) regulations have been in place in England which bans the placement of HFSS in prominent locations [26] and our results suggest that replacing HFSS items with healthier or non-food alternatives would be perceived positively by consumers. The newly announced NHS 10-year plan [57] implies that placement and promotion regulations will be repealed in favour of mandatory reporting where retailers must meet an obligatory target of healthy food sales by any means they choose (e.g. reformulating products, increasing promotions on healthy products or reducing prominent positioning of HFSS items). The results of the current study provide evidence that placing healthy and non-food items in prominent locations could be a useful strategy for retailers to use to achieve mandatory healthy sales targets.

Including people of low socioeconomic status in supermarket research is essential to ensure that resultant interventions and policy recommendations are representative of the population. Educational attainment is a useful measure of socioeconomic status [8]. Our study showed that level of educational attainment did not impact the direction or strength of the whole sample findings. This is similar to work by Stuber et al. (2024) who found no main effect across four store placement interventions when looking at socioeconomic status as a modifying factor of the interventions [23]. Our results are likely a result of our high number of highly educated participants. A systematic review of interventions exploring the association between availability and positioning of healthy foods on dietary behaviours, sales, and purchasing found inconsistent findings in studies in populations with a lower socioeconomic status likely as a result of low sample sizes and lack of power: only half of the twelve intervention studies demonstrated a positive association for health [19].

### Strengths and limitations

A strength of this study is the use of the simulated supermarket approach which did not require lengthy ethical processes including gatekeeper approval and data transfer agreements from real life supermarkets [29]. It also allowed for data collection from a large number of participants in a shorter time frame. Collecting data virtually during the global Covid-19 pandemic was also an advantage for this study, especially as many shoppers were more accustomed to online grocery shopping during the pandemic [58]. Nonetheless, it should be recognised that purchasing patterns also changed during the pandemic: there was an increase in the purchase of basic unprocessed ingredients as households spent time cooking from scratch at the same there was an increase in the purchase of more comfort or snack items at this time [59, 60]. These changes in purchasing patterns may have affected study results in phase 1. In addition, the 10-second viewing time used in the study may have encouraged more immediate or intuitive choices than would occur in some real-world shopping situations. However, a time limit was necessary to keep the task feasible for participants. Methodologically, our 2D simulated supermarket approach, coupled with the use of eye-tracking software to obtain visual attention data, meant that objective attention data could be collected whilst controlling the stimuli presented to participants. It also allowed us to focus purely on placement as the core construct of our study, removing all additional contextual factors that influence consumer’s attention to products including signage, price and presence of others. In particular, in the preferential looking task we were able to present carefully constructed healthy, unhealthy or non-food scenes next to one another which would not be possible in a real-world environment.

Whilst there are numerous studies indicating a strong correlation in results between studies using eye-tracking methods to observe free-viewing visual attention and studies using a mouse click method to determine interest in a product [50], our study demonstrated different results between these two tasks. Differences may be a result of setting bias (own home vs. lab). Furthermore, it may be that the nature of the tasks meant different concepts were being measured. Having to actively consider items that capture attention requires a decision-making process which is likely to be shaped by past experiences and personal preferences. Passively viewing the items, however, may require fewer decision-making processes and measure objective visual attention. Differences in results were also seen between the two eye-tracking experimental tasks which may be a result of how the participant perceived the task. In the journey task, participants were asked to simply view the simulated supermarket journeys whilst in the preferential looking task they were asked to try to remember what they viewed. Free-viewing the images without a clear purpose in the journey task (e.g. a stimulus driven task) might have meant that participants’ attention was less focused whereas aiming to memorise images [a goal driven task] in the preferential looking task may have sharpened this attention [39]. Furthermore, the journey task involved viewing five images for each product condition (15 in total) and the same images were used afterwards in the preferential looking task to show 90 dual combinations of the product conditions; task fatigue or order effects may therefore have occurred in the preferential looking task.

Future research would benefit from the inclusion of more groups of participants including fathers and young people to understand differences in attention and intentions to purchase products across a range of customers. We aimed to oversample participants of low socioeconomic status in our study by advertising in low-income areas and Sure Start centres but, despite this, most participants in both phases of the study held a Higher National Diploma or University Degree which will have affected results. To increase involvement of low socioeconomic groups in future research we may wish to utilise face-to-face recruitment and mobile eye tracking software to visit people in places that are most convenient to them.

## Conclusions

Women demonstrated a strong intention and preference for healthy foods to be placed in prominent locations in supermarkets to help them eat more healthily. There were mixed results regarding differences in attention between healthy, unhealthy and non-food items which suggests that supermarkets could place only healthy products in prominent locations with minimal commercial impact whilst supporting shoppers’ intention to buy healthy and public health policy. More research is needed to determine whether differences in socioeconomic status can affect shoppers’ attention to different food products.

## Supplementary Information

Supplementary Material 1.

Supplementary Material 2.

Supplementary Material 3.

Supplementary Material 4.

## Data Availability

The data for this study were collected by the research team. Anonymised data can be made available upon reasonable request to the corresponding author pending approval.

## Appendices

### Appendix 1: Example images from the simulated supermarket

*Store entrance images: healthy*,* non-food and unhealthy*.

*Aisle end images: healthy*,* non-food and unhealthy*.

*Checkout images: healthy*,* non-food and unhealthy*.

## Abbreviations

HFSS: High Fat, Sugar and Salt

## References

[1] Afshin A, Sur PJ, Fay KA, Cornaby L, Ferrara G, Salama JS, Mullany EC. Health effects of dietary risks in 195 countries, 1990–2017: a systematic analysis for the Global Burden of Disease Study 2017. Lancet. 2019;393:1958–72. 30954305 10.1016/S0140-6736(19)30041-8 PMC6899507

[2] Foldi M, Farkas N, Kiss S, Zadori N, Vancsa S, Szako L, Dembrovszky F. Obesity is a risk factor for developing critical condition in COVID-19 patients: A systematic review and meta-analysis. Obes Rev. 2020;21(10):e13095. 32686331 10.1111/obr.13095 PMC7404429

[3] Office for Improvement and Disparities. Obesity Profile: short statistical commentary July 2022. London: UK. Gov.UK; 2022.

[4] NHS Digital. National Child Measurement Programme, school year 2023/2024. London: UK. Gov.UK; 2024.

[5] Tony Blair Institute for Global Change. Unhealthy Numbers: The Rising Cost of Obesity in the UK. 2023. Unhealthy Numbers: The Rising Cost of Obesity in the UK. Accessed 28 Oct 2025.

[6] Rauber F, da Costa Louzada ML, Steele EM, de Rezende LFM, Millett C, Monteiro CA, Levy RB. Ultra-processed foods and excessive free sugar intake in the UK: a nationally representative cross-sectional study. BMJ Open. 2019;9:e027546. 10.1136/bmjopen-2018-027546. 10.1136/bmjopen-2018-027546 PMC6830631 31662351

[7] MRC Epidemiology Unit. National Diet and Nutrition Survey 2019–2023: Key findings on fruit, vegetable, fibre, saturated fat and free sugars intake. University of Cambridge / UK Data Service. 2025.

[8] Vogel C, Ntani G, Inskip H, Barker M, Cummins S, Cooper C, Moon G, Baird J. Education and the Relationship Between Supermarket Environment and Diet. Am J Preventative Med. 2016;51(2):e27–34. 10.1016/j.amepre.2016.02.030 PMC4959574 27067035

[9] Monteiro CA, Cannon G, Levy RB, Moubarac JC, Jaime P, Martins AP, Nova Group. Ultra-processed foods: what they are and how to identify them. Public Health Nutr. 2019;22(5):936–41. 30744710 10.1017/S1368980018003762 PMC10260459

[10] Dimbleby H. National Food Strategy independent review: The plan. 2021. London.

[11] Kantar World Panel. Grocery Market Share. 2025. Grocery Market Share - Kantar Accessed 5 Mar 2025.

[12] Thorndike AN, Sunstein CR. Obesity prevention in the supermarket—choice architecture and the Supplemental Nutrition Assistance Program. Am J Public Health. 2017;107(10):1582. 28902555 10.2105/AJPH.2017.303991 PMC5607683

[13] Department for Environment Food and Rural Affairs. Food Statistics in your pocket 2017: Food Chain. York, UK: 2017.

[14] Ball S. Oct. Weighing the impact of HFSS laws. 2023. Weighing the impact of HFSS laws Accessed 28 2025.

[15] The Food Foundation. The Broken Plate. 2025 TFF_The Broken Plate 2025.pdf Accessed 28 Oct 2025.

[16] Vogel C, Piernas C. The Retail Food Environment. In: Evans CEL, editor. Transforming Food Environments. CRC; 2022. pp. 63–78.

[17] Obesity Health Alliance. Out of Place – How unhealthy supermarket promotions are bad for our wallets and our waistlines. 2018 https://obesityhealthalliance.org.uk/2018/11/19/place-unhealthy-supermarket-promotions-bad-wallets-waistlines Accessed 28 Oct 2025.

[18] Thorndike AN. Healthy choice architecture in the supermarket: Does it work? Soc Sci Med. 2020;266:113459. 33127172 10.1016/j.socscimed.2020.113459

[19] Shaw SC, Ntani G, Baird J, Vogel CA. A systematic review of the influences of food store product placement on dietary-related outcomes. Nutr Rev. 2020;78(12):1030–45. 32483615 10.1093/nutrit/nuaa024 PMC7666915

[20] Huitink M, Poelman MP, Seidell JC, Kuijper LD, Hoekstra T, Dijkstra SC. Can healthy checkout counters in supermarkets promote healthier food choices? Two real-life experiments in supermarkets in a disadvantaged urban area in the Netherlands. BMC Public Health. 2020;20:542. 32316936 10.1186/s12889-020-08608-6 PMC7171819

[21] Vogel C, Crozier S, Penn-Newman D, Ball K, Moon G, Lord J, Coper C, Baird J. Altering product placement to create a healthier layout in supermarkets: Outcomes on store sales, customer purchasing, and diet in a prospective matched controlled cluster study. PLoS Med. 2021;18(9):e1003729. 34491999 10.1371/journal.pmed.1003729 PMC8423266

[22] Piernas C, Harmer G, Jebb SA. Removing seasonal confectionery from prominent store locations and purchasing behaviour within a major UK supermarket: Evaluation of a nonrandomised controlled intervention study. PLoS Med. 2022;19(3):e1003951. 35324903 10.1371/journal.pmed.1003951 PMC8946674

[23] Stuber JM, Beulens KWJ, Ayala GX, Crozier SR, Dijkstra SC, Lin SF, Mackenbach JD. Can nudge interventions targeting healthy food purchases in real-world grocery stores reduce diet-related health disparities? A pooled analysis of four controlled studies. Internation J Behav Nutr Phys Activity. 2024;21:137. 10.1186/s12966-024-01687-3 PMC11616344 39627771

[24] Falbe J, Marinello S, Wolf EC, Solar S, Powell LM. Food environment after implementation of a healthy checkout policy. JAMA Netw Open. 2024;7(8):e2421731. 39115848 10.1001/jamanetworkopen.2024.21731 PMC11310826

[25] Adams J, Mytton O, White M, Monsivais P. Why Are Some Population Interventions for Diet and Obesity More Equitable and Effective Than Others? The Role of Individual Agency. PLoS Med. 2016;13(4):e1001990. 27046234 10.1371/journal.pmed.1001990 PMC4821622

[26] UK Government. The Food (Promotion and Placement) (England) Regulations 2021. 10.1186/s12916-024-03720-5 PMC11539601 39506702

[27] The Scottish Government. Restriction promotion of food and drink high in fat, sugar or salt: consultation analysis. Gov Scot. 2025.

[28] Welsh Government. The Food (Promotion and Presentation) (Wales) Regulations 2025: Implementation Guidance. Gov.Wales. 2025.

[29] Vogel C, Dijkstra C, Huitink M, et al. Real-life experiments in supermarkets to encourage healthy dietary-related behaviours: opportunities, challenges and lessons learned. Int J Behav Nutr Phys Activity. 2023;20(1):73. 10.1186/s12966-023-01448-8 PMC10280909 37340326

[30] Cullerton K, Adams J, Forouhi NG, Francis O, White M. Avoiding conflicts of interest and reputational risks associated with population research on food and nutrition: the Food Research risK (FoRK) guidance and toolkit for researchers. BMJ. 2024;384:e077908. 38286473 10.1136/bmj-2023-077908 PMC10823375

[31] Borgmeier I, Westenhoefer J. Impact of different food label formats on healthiness evaluation and food choice of consumers: a randomized-controlled study. BMC Public Health. 2009;9:184. 19523212 10.1186/1471-2458-9-184 PMC2702386

[32] Talati Z, Pettigrew S, Kelly B, Ball K, Dixon H, Shilton T. Consumers’ responses to front-of-pack labels that vary by interpretive content. Appetite. 2016;101:205–13. 26970293 10.1016/j.appet.2016.03.009

[33] Machín L, Aschemann-Witzel J, Curutchet MR, Giménez A, Ares G. Does front-of-pack nutrition information improve consumer ability to make healthful choices? Appetite. 2018;121:55–62. 29102533 10.1016/j.appet.2017.10.037

[34] Egnell M, Talati Z, Hercberg S, Pettigrew S, Julia C. Objective understanding of front-of-pack nutrition labels: an international comparative experimental study. Nutrients. 2018;10(10):1542. 30340388 10.3390/nu10101542 PMC6213801

[35] Sorensen H. Inside the mind of the shopper: the science of retailing. Upper Saddle River (NJ): Pearson Education; 2009.

[36] Vermeir I, Roose G. Visual design cues and their influence on consumer behaviour: a review. J Econ Psychol. 2020;79:102256.

[37] Food Standards Agency. The psychology of consumer behaviour: a review of evidence. London: FSA; 2020.

[38] Maynard OM, McClernon FJ, Oliver JA, Munafo MR. Using Neuroscience to Inform Tobacco Control Policy. Nicotine Tob Res. 2018;21(6):739–46. 10.1093/ntr/nty057 PMC6528159 29590482

[39] Bialkova S, Gruner KG, van Trijp H. From desktop to supermarket shelf: Eye-tracking exploration on consumer attention and choice. Food Qual Prefer. 2020;81:103839.

[40] Gidlof K, Anikin A, Lingonblad M, Wallin A. Looking is buying. How visual attention and choice are affected by consumer preferences and properties of the supermarket shelf. Appetite. 2017;116:29–38. 28433775 10.1016/j.appet.2017.04.020

[41] Clement J, Kristensen T, Grønhaug K. Understanding consumers’ in-store visual perception: The influence of package design features on visual attention. J Retailing Consumer Serv. 2013;20(2):234–39.

[42] Kim N, Lee H. Assessing consumer attention and arousal using eye-tracking technology in virtual retail environment. Front Psychol. 2021;12:665658. 34434136 10.3389/fpsyg.2021.665658 PMC8380820

[43] The Grocer. Eye-tracking study shows power of gondola ends for HFSS brands. 2022. Eye-tracking study shows power of gondola ends for HFSS brands | News | The Grocer Accessed 28 Oct 2025.

[44] Pieters R, Warlop L. Visual attention during brand choice: the impact of time pressure and task motivation. Int J Res Mark. 1999;16(1):1–16.

[45] Food Standards Agency. The 2014 Food and You survey. London, 2014.

[46] Singh S, Cordeiro A, Epel E. Association between maternal eating and young child feeding in a community sample. BMC Pregnancy Childbirth. 2023;23(470). 10.1186/s12884-023-05786-0. 10.1186/s12884-023-05786-0 PMC10290385 37355578

[47] Black C, Ntani G, Inskip H, Cooper C, Cummins S, Moon G, Baird J. Measuring the healthfulness of food retail stores: variations by store type and neighbourhood deprivation. Int J Behav Nutr Phys Activity. 2014;11(1):articlenumber69. 10.1186/1479-5868-11-69. 10.1186/1479-5868-11-69 PMC4132210 24884529

[48] Monteiro CA, Cannon G, Lawrence M, Costa Louzada ML, Pereira Machado P. Ultra-processed foods, diet quality, and health using the NOVA classification system. Rome, FAO. 2019.

[49] Belteki Z, Hessels RS, Junge CMM, Kemner C, van den Boomen C. How infants direct their gaze to faces in the presence of other objects: The development of face preference between 4 and 7 months after birth. Infancy. 2024;30(1):e12633. 10.1111/infa.12633 39551722

[50] Henderson JM, Hollingworth A. High-level scene perception. Ann Rev Psychol. 1999;50:243–71. 10074679 10.1146/annurev.psych.50.1.243

[51] Braun V, Clarke V. Using thematic analysis in psychology. Qualitative Res Psychol. 2006;3(2):77–101.

[52] StataCorp. Stata: Release 17. Statistical Software. College Station, TX: Stata Corp LLC; 2025.

[53] The Food, Farming and Countryside Commission The Food Conversation. 2023. The Food Conversation. Accessed 7 Oct 2025.

[54] Dhuria P, Lawrence W, Crozier S, Cooper C, Baird J, Vogel C. Women’s perceptions of factors influencing their food shopping choices and how supermarkets can support them to make healthier choices. BMC Public Health. 2021;21:1070. 34090410 10.1186/s12889-021-11112-0 PMC8178895

[55] Ford A, Eadie D, Adams J, Adamson A, White M, Stead M. Parents’ and carers’ awareness and perceptions of UK supermarket policies on less healthy food at checkouts: A qualitative study. Appetite. 2020;147:104541. 31778731 10.1016/j.appet.2019.104541

[56] Muir S, Dhuria P, Roe E, Lawrence W, Baird J, Vogel C. UK government’s new placement legislation is a ‘good first step’: a rapid qualitative analysis of consumer, business, enforcement and health stakeholder perspectives. BMC Med. 2023;21(1):33. 10.1186/s12916-023-02726-9 PMC9878939 36703194

[57] UK Government. Fit for the future: 10 year health plan for England. Gov. UK. 2025.

[58] Office for National Statistics. Online shopping, UK. 2022. 2022. Available from: https://www.ons.gov.uk/businessindustryandtrade/retailindustry/articles/onlineshoppinguk/2022 (ons.gov.uk in Bing).

[59] Food Standards Agency. *Food and You 2*: Wave 1. Food Standards Agency. 2021. https://www.food.gov.uk/research/food-and-you/food-and-you-2-wave-1 (food.gov.uk in Bing).

[60] Ammar A, Brach M, Trabelsi K, et al. Effects of COVID-19 home confinement on eating behaviour and physical activity: Results of the ECLB-COVID19 international online survey. Nutrients. 2020;12(6):1583. 32481594 10.3390/nu12061583 PMC7352706

