# Supplementary material for: Women intend to buy, and attend more to, healthy foods in supermarkets: attention to product placement observed in women in a 2-dimensional simulated supermarket environment
Source: BMC Public Health. 2026 May 18;26:2131. doi: 10.1186/s12889-026-27679-5 (PMC13362013; doi:10.1186/s12889-026-27679-5)
Supplement: Supplementary file 1 — Supplementary Material 1. [file 12889_2026_27679_MOESM1_ESM.docx]

Supplementary file 1: Products shown in each display image

| Location | Healthy | Unhealthy | Non-Food |
| --- | --- | --- | --- |
| Entrance  (4 items) | **Bulk buy drinks and cereals**  -Tea  -Coffee  -Oats  -No added sugar muesli | **Bulk buy drinks and cereals**  -Frosted Flakes  -Choco Puffs  -Cola  -Orange Fizz Drink | **Bulk items**  -Bathroom Spray  -Bleach  -Quilted Toilet Roll  -Basic Toilet Roll |
| End of aisle 1  (11 Items) | **Snacking (multipacks and sharing)**  -Monkey nuts  -Raw Red nuts  -Brazil nuts  -Jumbo Cashew Nuts  -Blanched Almonds  -Chia Seeds  -Walnuts  -Raisins  -Dried Pineapple  -Dried Mango  -Orchard Prunes | **Snacking (multipacks and sharing)**  -Digestive biscuits  -Jaffa Cakes  -Chocolate biscuit bars  -Malted chocs  -Chewy sweets  -Candy Coated buttons  -Chocolate fingers  -Animal biscuits  -Crisp Snacks  -Teddy faces  -Hoop crisps | **Cleaning Products**  -Washing up liquid 1  -Washing up liquid 2  -Dishmop  -Dishmop refills  -Surface cleaner  -Anti-bac wipes  -Floor cleaner  -Oven Cleaner  -Dishwasher tablets  -All-in-1 dishwasher  -Kitchen Roll |
| End of aisle 2  (11 Items) | **Meal staples**  -White long grain rice  -Wholewheat Penne  -Spaghetti  -Fusilli  -Dried Lentils  -Tahini  -Basmati Rice  -Brown rice  -Dried oregano  -Garlic Powder  -Paprika | **Meal staples**  -Pasta in sauce  -Flavoured noodles 1  -Soup cups  -Baked beans  -Tomato soup  -Spaghetti Hoops  -Bolognaise Sauce  -Carbonara Sauce  -Chilli con carne sauce  -Cheesy Enchilada  -Flavoured noodles 2 | **Toiletries**  -Shampoo brand 1  -Conditioner brand 1  -Shampoo brand 2  -Conditioner brand 2  -Shampoo brand 3  -Conditioner brand 3  -Moisturiser  -Shower Gel  -Shaving Cream  -Bio-oil  -Hand Cream |
| End of aisle 3  (11 Items) | **Breakfast items**  -Flaxseeds brand 1  -Flaxseeds brand 2  -12 Eggs  -6 Eggs  -Long-life semi milk  -Long-life skim milk  -Orange Juice  -Tinned Peach Slices  -Apple Juice  -Wheat Biscuits  -Bitesize Wheat Biscuits | **Breakfast items**  -Berry cereal bars  -Walk flapjacks  -Mountain Cereal bars  -Breakfast biscuit bars  -Chewy granola bar  -Crispy crunch granola  -Granulated sugar  -Cookie Cereal  -Milkshake powder  - Hoops Cereal  -Crazy Choc cereal | **Hygiene Products**  -Baby Dry Nappies  -Baby Wipes  -Nappy Pants  -Baby Powder  -Baby Oil  -Ladies Razors  -Tampons  -Sanitary pads brand 1  -Sanitary pads brand 2  -Deodorant  -Body Spray |
| Checkout  (10 items) | **Grab and Go Snacks**  -Dried Apricots  -Dried Mango  -Dried Strawberries  -Raisins  -Unsalted Cashews  -Four Seed snack  -Apple Rice Cakes  -Unsalted Peanuts  -Chia shots  -Canned water | **Grab and Go Snacks**  -Chocolate Egg  - Choc dip with sticks  -Mints  -Milk chocolate bar  -Caramel Choc bar  -Chunky wafer bar  -Bubbly chocolate bar  -Marshmallow stick  -Red Energy Drink  -E Energy Drink | **Grab and Go Items**  -Ibuprofen  -Paracetamol  -Hand Sanitiser  -Vaseline lip balm  -Football cards  -Colouring books  -Colouring pencils  -Large Water Bottle  -Small Water Bottle  -Face Masks |
