# Supplementary material for: Women intend to buy, and attend more to, healthy foods in supermarkets: attention to product placement observed in women in a 2-dimensional simulated supermarket environment
Source: BMC Public Health. 2026 May 18;26:2131. doi: 10.1186/s12889-026-27679-5 (PMC13362013; doi:10.1186/s12889-026-27679-5)
Supplement: Supplementary file 2 — Supplementary Material 2. [file 12889_2026_27679_MOESM2_ESM.pdf]

Phase 1 Virtual Supermarket – Hosted on Qualtrics

Start of Block: CONSENT

CONSENT FORM

Please select ALL boxes in order to continue with your participation in this study. When you have done this please click the arrow at the bottom of the screen to go to the next page.

	Please select all (1)
I have read and understood the information sheet provided by the research team (Version 2, 10/12/20) and have had the opportunity to ask questions about the study (1)	○
I agree to take part in this research project and agree for my data to be used for the purpose of this study (2)	○
I understand that my participation is voluntary and I may withdraw at any time and for any reason without my participation rights being affected (3)	○
I understand that the research team will be able to link my survey responses to my identity, so that they know who has completed the survey and who is eligible for an Amazon voucher (4)	○
I have been given contact details for the researchers and informed that I can contact them if I have any questions at any point before, during, or after I participate in the experiment (5)	○
I understand that I will not be directly identified in any reports of the research (7)	○
I understand that my personal information collected about me such as my postcode will not be shared with anyone beyond the study team (8)	○

End of Block: CONSENT

Start of Block: DEMOGRAPHICS

DEMOGRAPHICS FORM

We'd like to collect some data so we know who our participants are for research purposes. Please answer ALL the questions. When you have done this and you are ready, please click the button at the bottom of the screen to start the experiment.

PLEASE NOTE - ALL THIS INFORMATION WILL BE KEPT CONFIDENTIAL AND STORED SECURELY. IT WILL NOT BE SHARED WITH ANYONE OUTSIDE THE RESEARCH TEAM AND IS FOR STUDY PURPOSES ONLY. NONE OF THE CONTACT DETAILS YOU GIVE WILL BE SHARED EITHER.

My participant ID number (provided by the research team) is:

____

My initials are:

____

My postcode:

____

Gender

I am:

- ◯ Male (1)
- ◯ Female (2)
- ◯ I define myself in another way (3) ____

My age in years:

____

My highest level of qualification achieved:

- ◯ None (2)
- ◯ GCSE D or below/BTEC level 1/NVQ level 1 (3)
- ◯ GCSE A*-C/BTEC level 2/NVQ level 2 (4)
- ◯ A Level/BTEC level 3/NVQ level 3 (7)
- ◯ Higher National Diploma (8)
- ◯ Degree or above (9)
- ◯ Other (please specify) (5) ____

End of Block: DEMOGRAPHICS

Start of Block: PHASE 1 INSTRUCTIONS

Phase 1 instructions
After clicking the button below to move onto the next page, you will start the experiment.

The experiment consists of 2 phases. Each phase will contain 3 'journeys' through the virtual supermarket. In the first phase we are interested in products that catch your attention. In the second phase we are interested in products that you would purchase if this were a shopping trip.

For this first phase, please **click on any products that catch your attention.** When you have clicked, a blue dot will appear. You can select **UP TO 10** products. If you wish to remove a blue dot, you can click on it and it will disappear. If you click on more than 10, the earlier ones will start to disappear.

Each image will be displayed for 10 seconds.

In between each of these images will be a filler image of a supermarket aisle. You do not have to do anything for these images and they will only be displayed for a short amount of time (2 seconds).

Once you start the experiment you will not be able to pause it and you must complete it all in one go so please do not start until you are ready (It will take around 10 minutes in total).

End of Block: PHASE 1 INSTRUCTIONS

Start of Block: UNHEALTHY1

Timer1 Timing
First Click (1)
Last Click (2)
Page Submit (3)
Click Count (4)

---

entrance_Unhealthy1

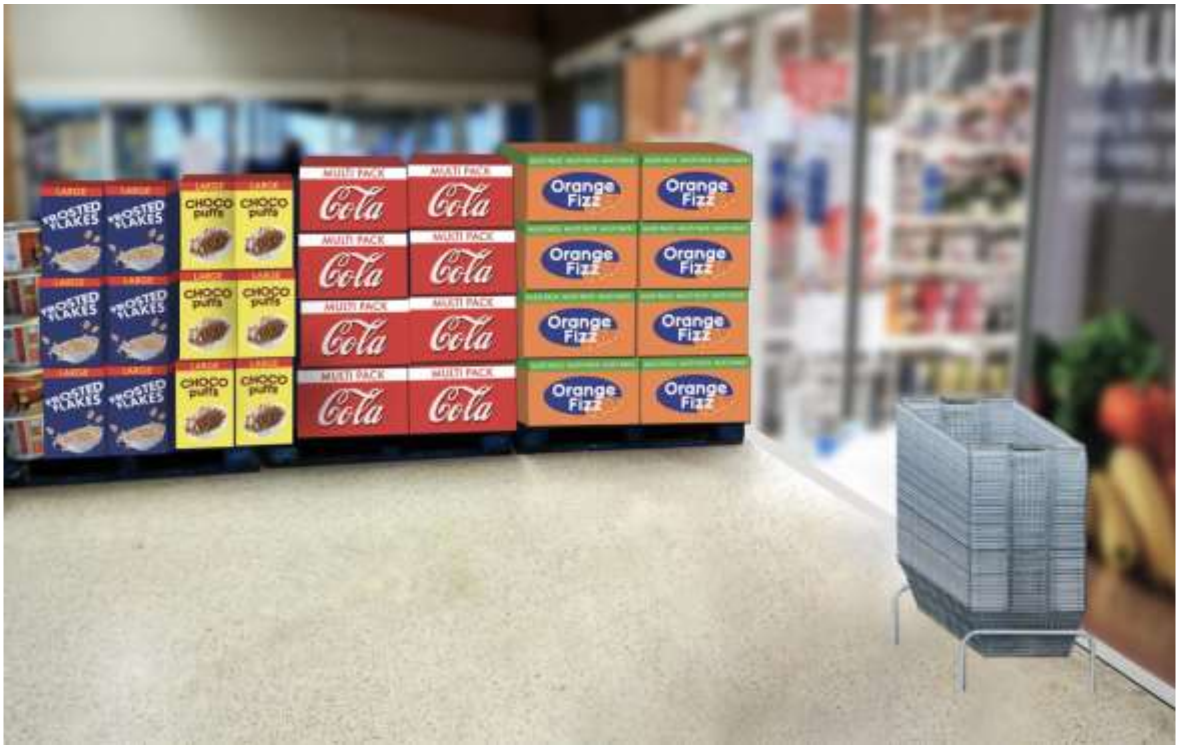

Page Break

timer_2 Timing
First Click (1)
Last Click (2)
Page Submit (3)
Click Count (4)

blurred_aisle_1

Page Break

Timer_3 Timing
First Click (1)
Last Click (2)
Page Submit (3)
Click Count (4)

EOA_1_unhealthy1

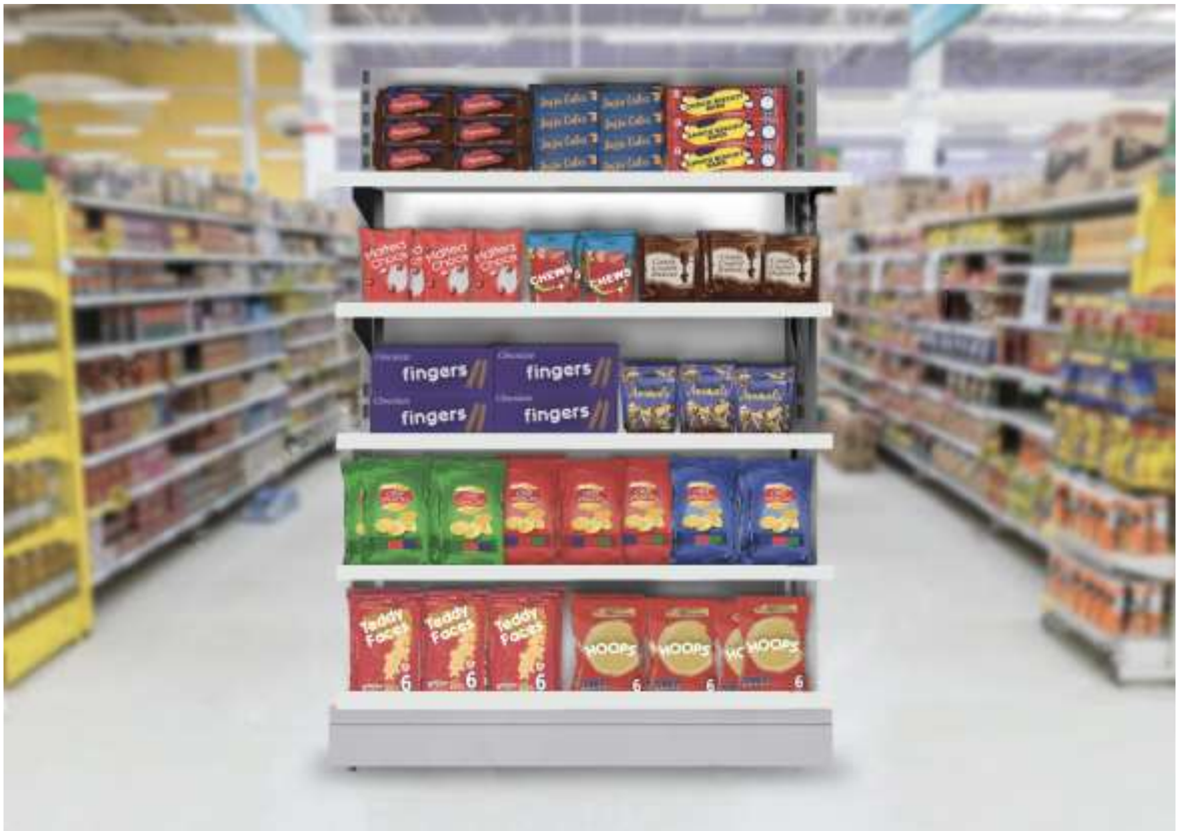

Page Break

Timer_4 Timing
First Click (1)
Last Click (2)
Page Submit (3)
Click Count (4)

---

blurred_aisle_2

Page Break

Timer_5 Timing
First Click (1)
Last Click (2)
Page Submit (3)
Click Count (4)

EOA_2_unhealthy1

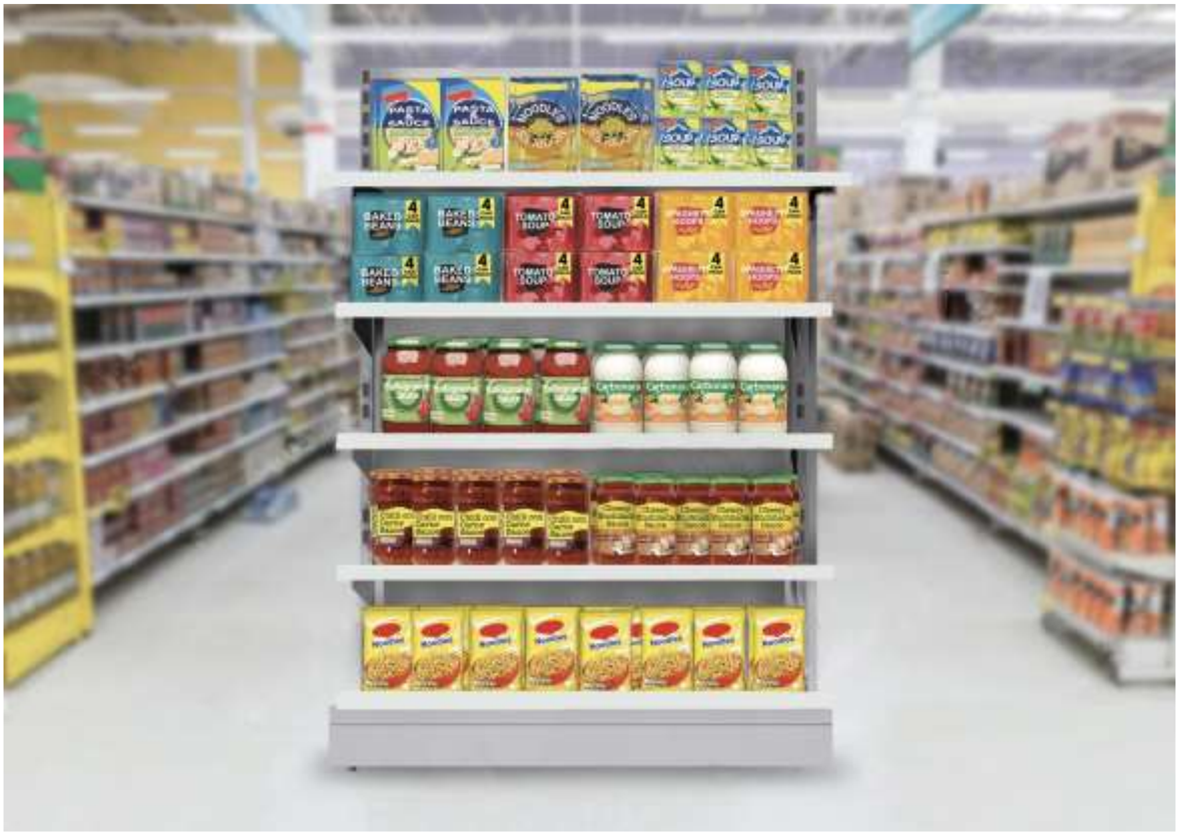

Page Break

Timer_6 Timing
First Click (1)
Last Click (2)
Page Submit (3)
Click Count (4)

blurred_aisle_3

Page Break

Timer_7 Timing
First Click (1)
Last Click (2)
Page Submit (3)
Click Count (4)

EOA_3_unhealthy1

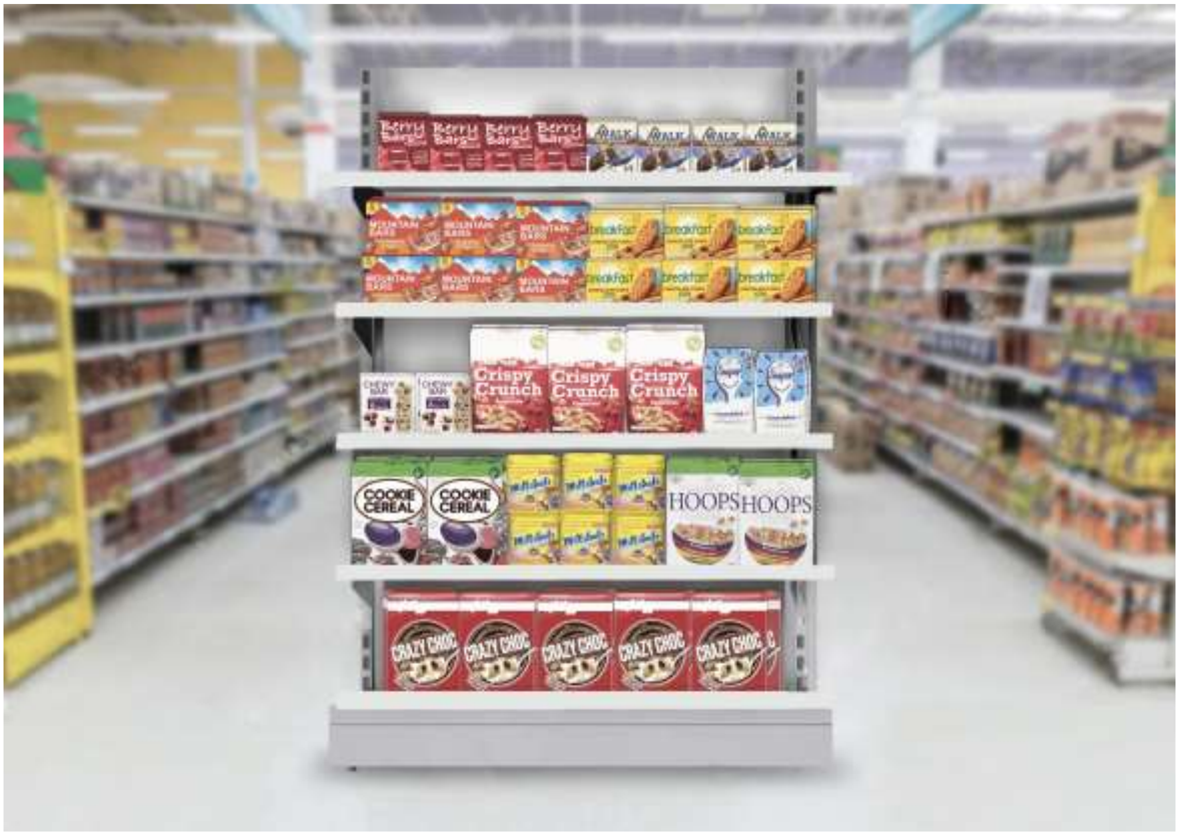

Page Break

Timer_8 Timing
First Click (1)
Last Click (2)
Page Submit (3)
Click Count (4)

---

blurred_aisle_4

Page Break

Timer_9 Timing
First Click (1)
Last Click (2)
Page Submit (3)
Click Count (4)

Unhealthy Checkout1

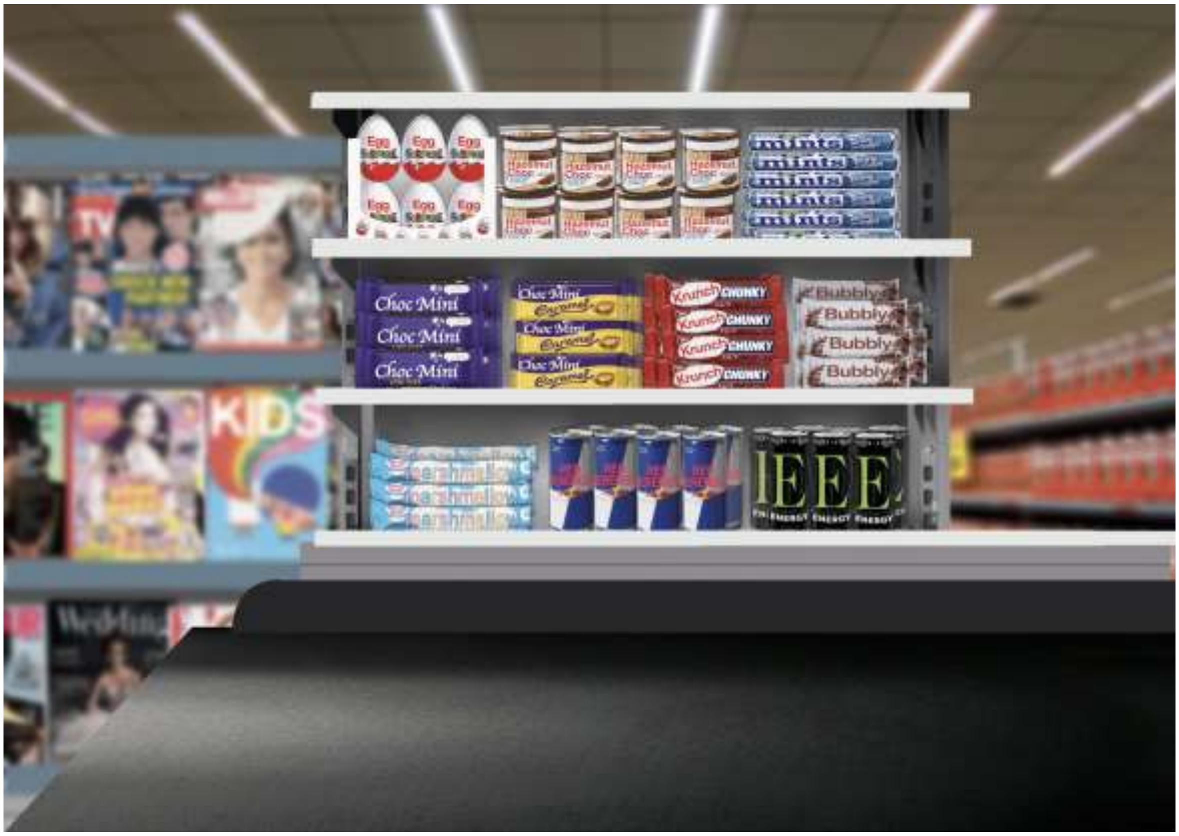

End of Block: UNHEALTHY1

Start of Block: End of Journey 1

timer1 Timing
First Click (1)
Last Click (2)
Page Submit (3)
Click Count (4)

---

endofjourney1
You have completed store journey 1 of 6.   There will now be a 20 second break before the next journey.   Thank you :)

---

calm_image1

End of Block: End of Journey 1

Start of Block: HEALTHY1

timer1 Timing
First Click (1)
Last Click (2)
Page Submit (3)
Click Count (4)

Entrance_healthy1

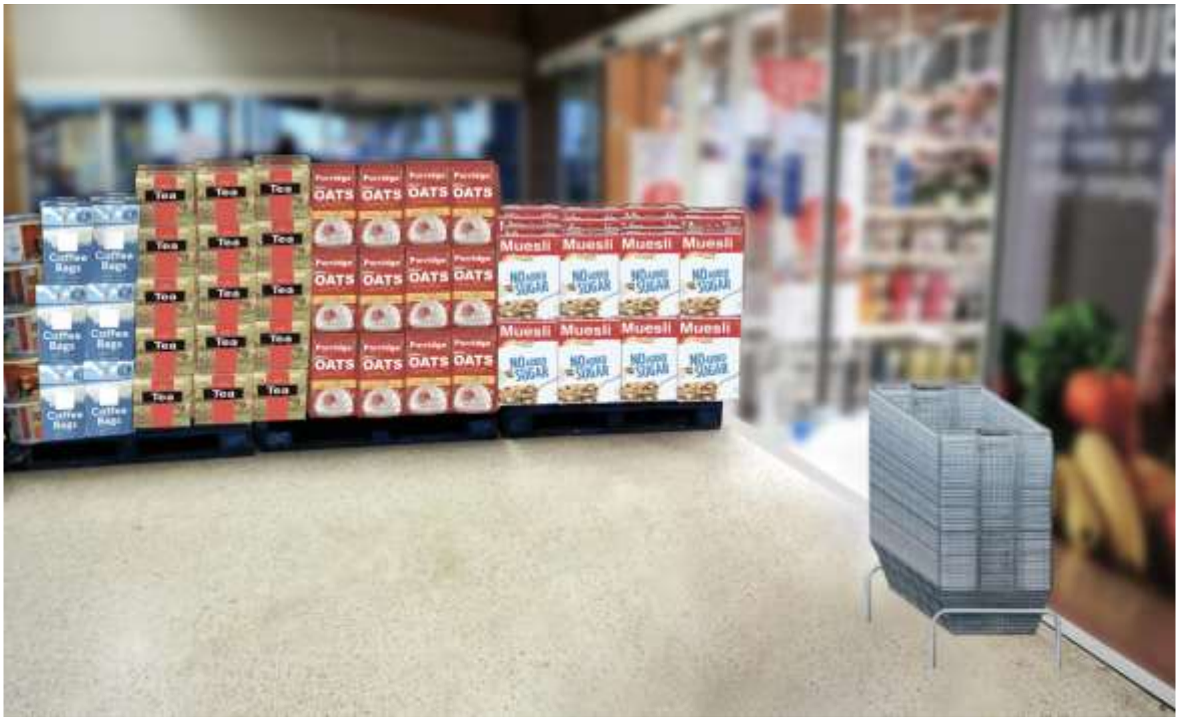

Page Break

timer2 Timing
First Click (1)
Last Click (2)
Page Submit (3)
Click Count (4)

blurred_aisle1

Page Break

timer3 Timing
First Click (1)
Last Click (2)
Page Submit (3)
Click Count (4)

EOA_Healthy1

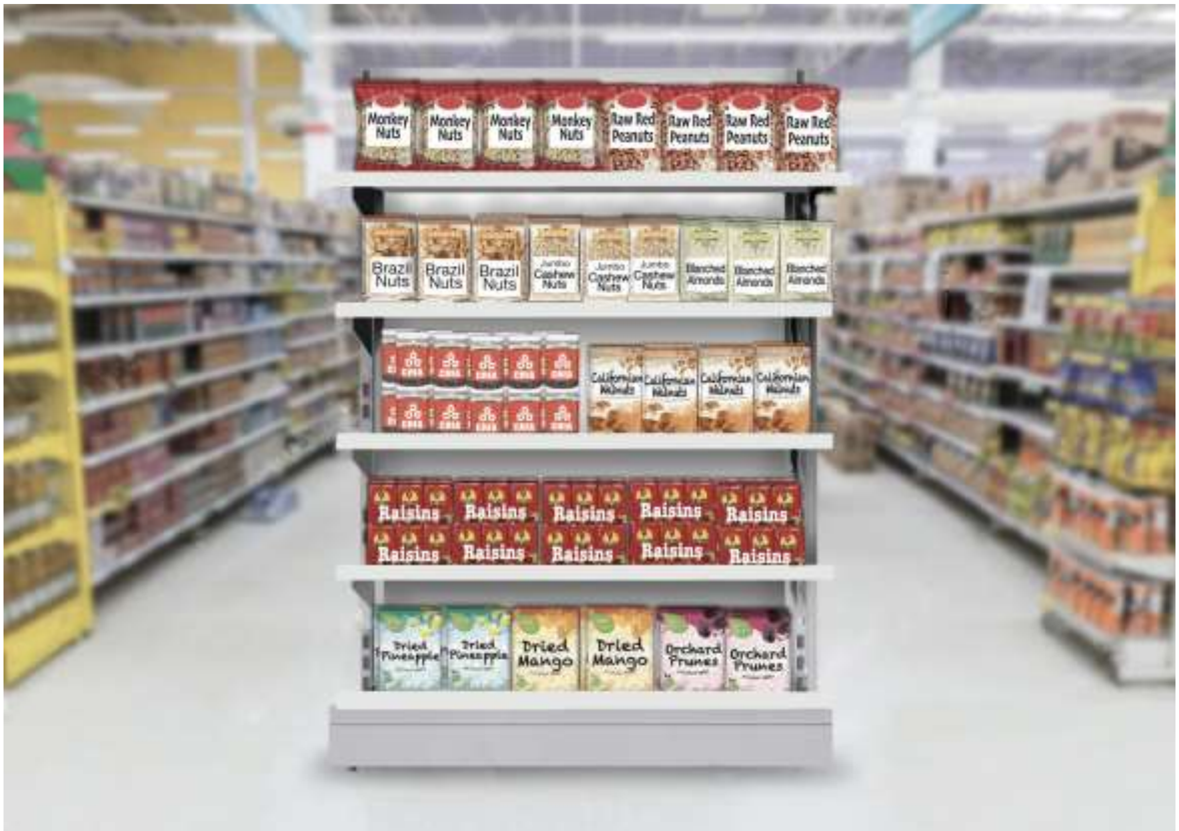

Page Break

timer4 Timing
First Click (1)
Last Click (2)
Page Submit (3)
Click Count (4)

---

blurred_aisle2

Page Break

timer5 Timing
First Click (1)
Last Click (2)
Page Submit (3)
Click Count (4)

EOA_Healthy2

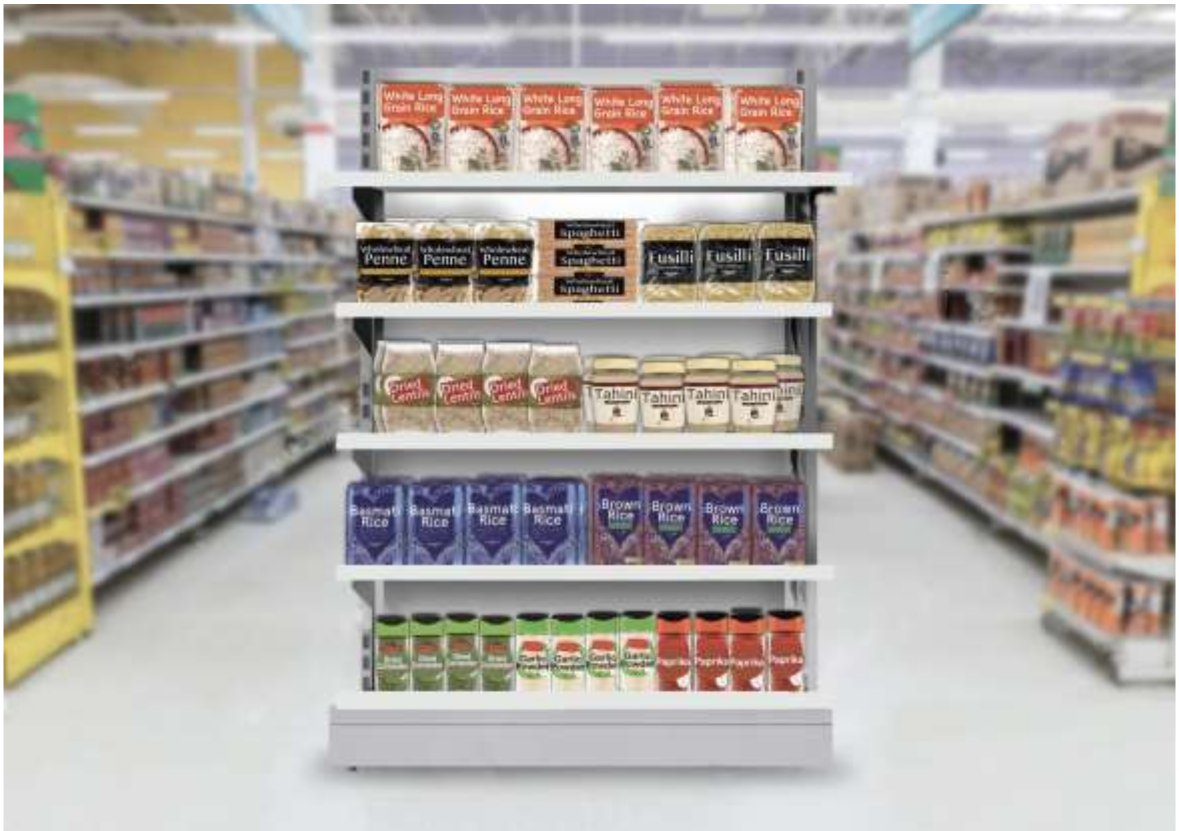

Page Break

timer6 Timing
First Click (1)
Last Click (2)
Page Submit (3)
Click Count (4)

---

blurred_aisle3

Page Break

timer7 Timing
First Click (1)
Last Click (2)
Page Submit (3)
Click Count (4)

EOA_Healthy3

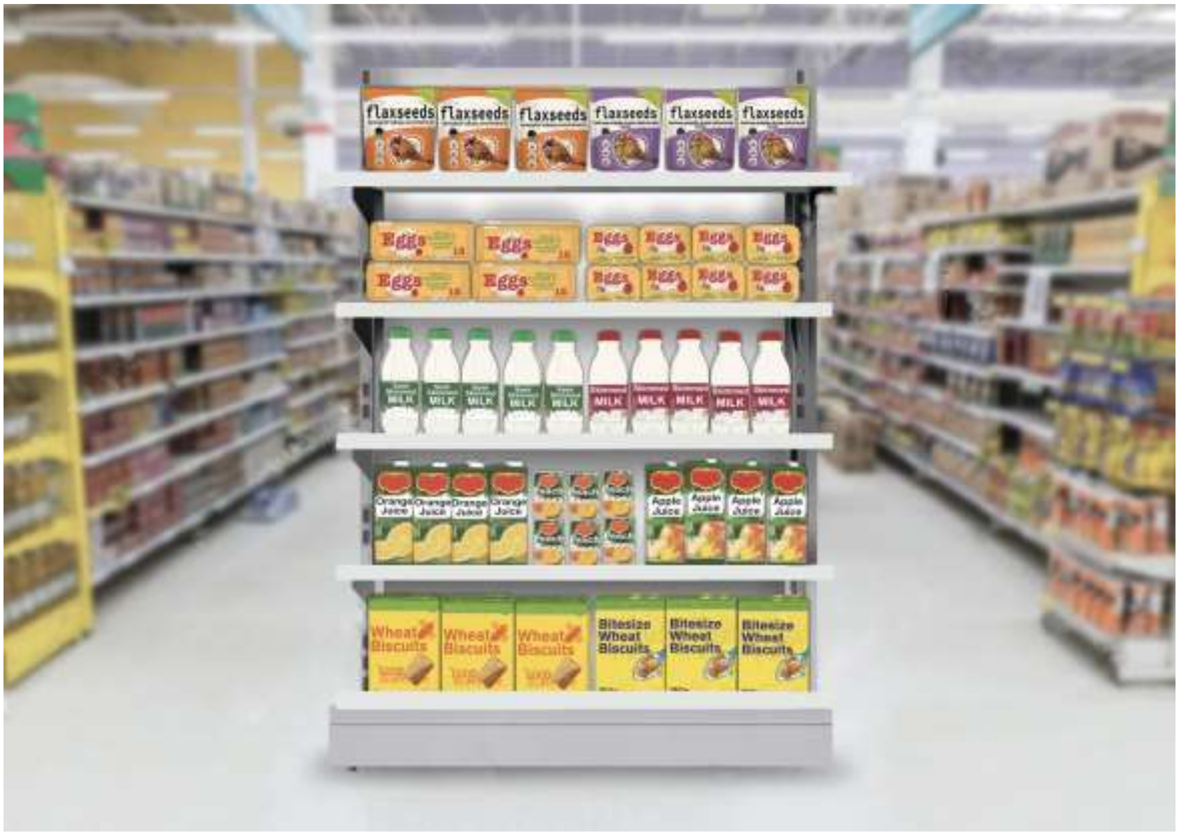

Page Break

timer8 Timing
First Click (1)
Last Click (2)
Page Submit (3)
Click Count (4)

blurred_aisle4

Page Break

timer9 Timing
First Click (1)
Last Click (2)
Page Submit (3)
Click Count (4)

---

checkout_healthy1

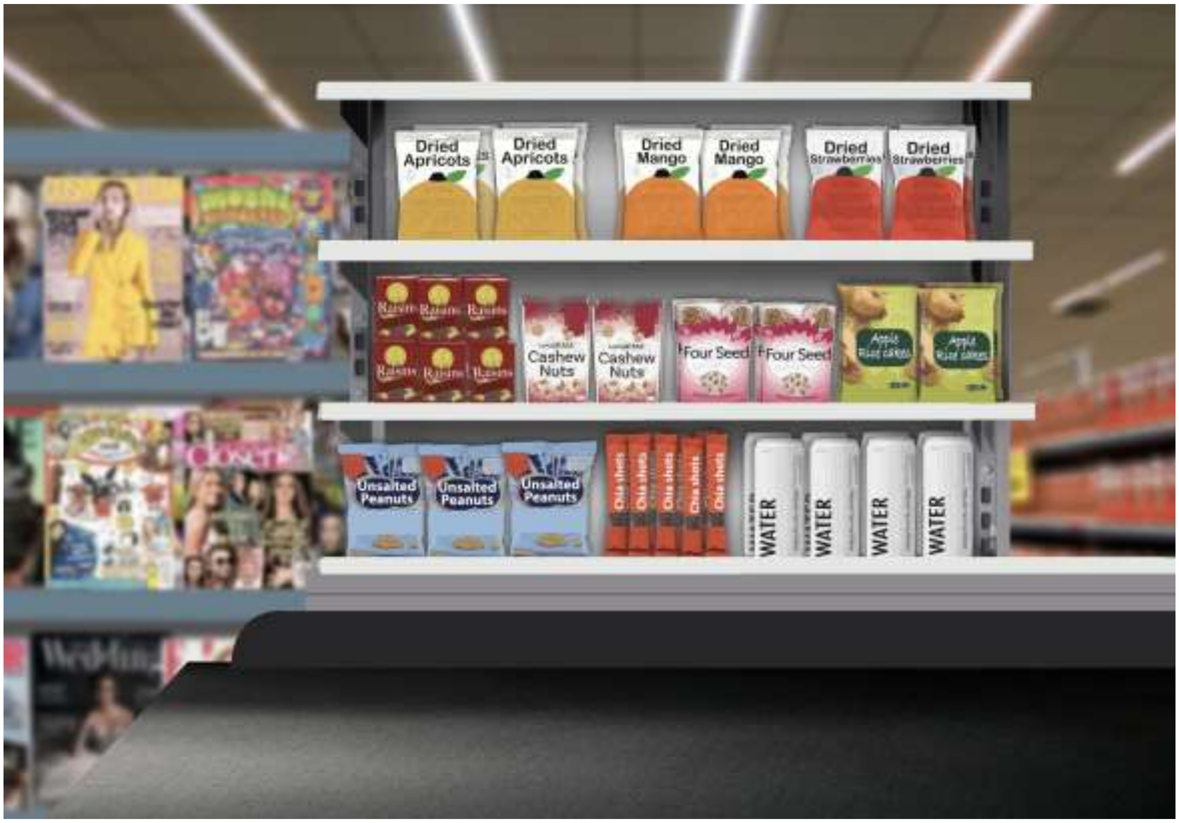

End of Block: HEALTHY1

Start of Block: End of journey 2

timer1 Timing
First Click (1)
Last Click (2)
Page Submit (3)
Click Count (4)

---

endofjourney2
You have completed store journey 2 of 6.   There will now be a 20 second break before the next journey.   Thank you :)

---

calm_image2

End of Block: End of journey 2

Start of Block: NON FOOD1

TImer1 Timing
First Click (1)
Last Click (2)
Page Submit (3)
Click Count (4)

Entrance_nonfood1
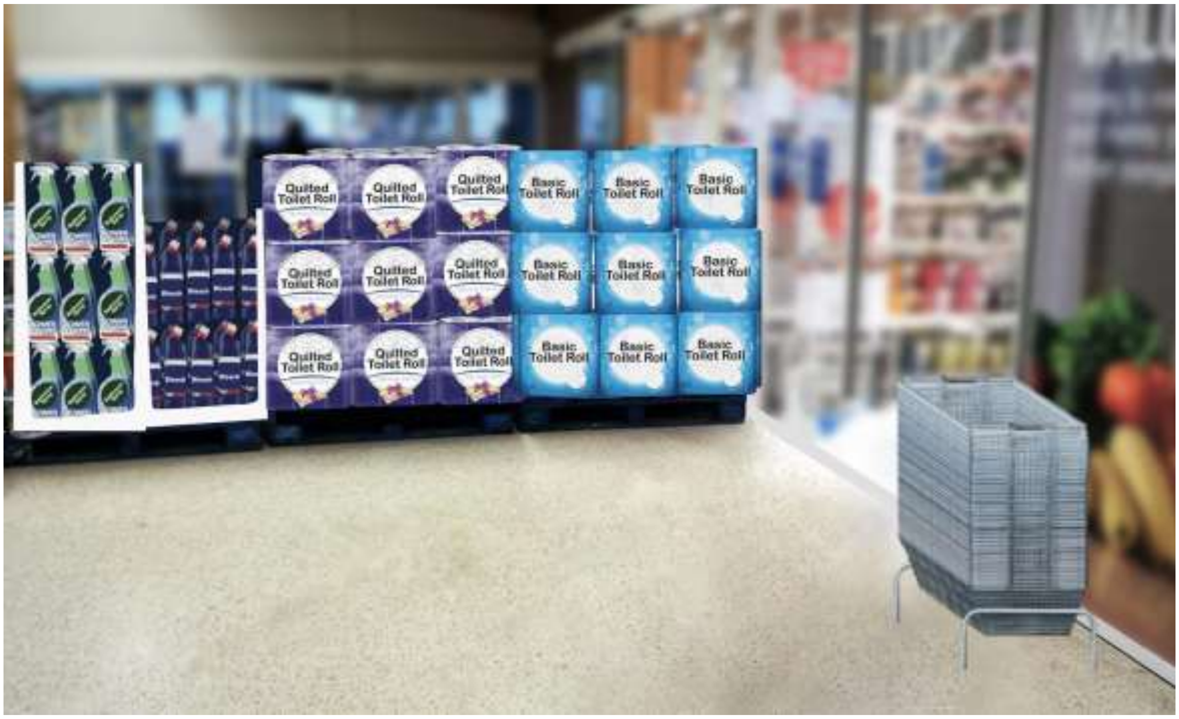

Page Break

Timer2 Timing
First Click (1)
Last Click (2)
Page Submit (3)
Click Count (4)

blurred_aisle_1

Page Break

Timer3 Timing
First Click (1)
Last Click (2)
Page Submit (3)
Click Count (4)

EOA1_nonfood_1

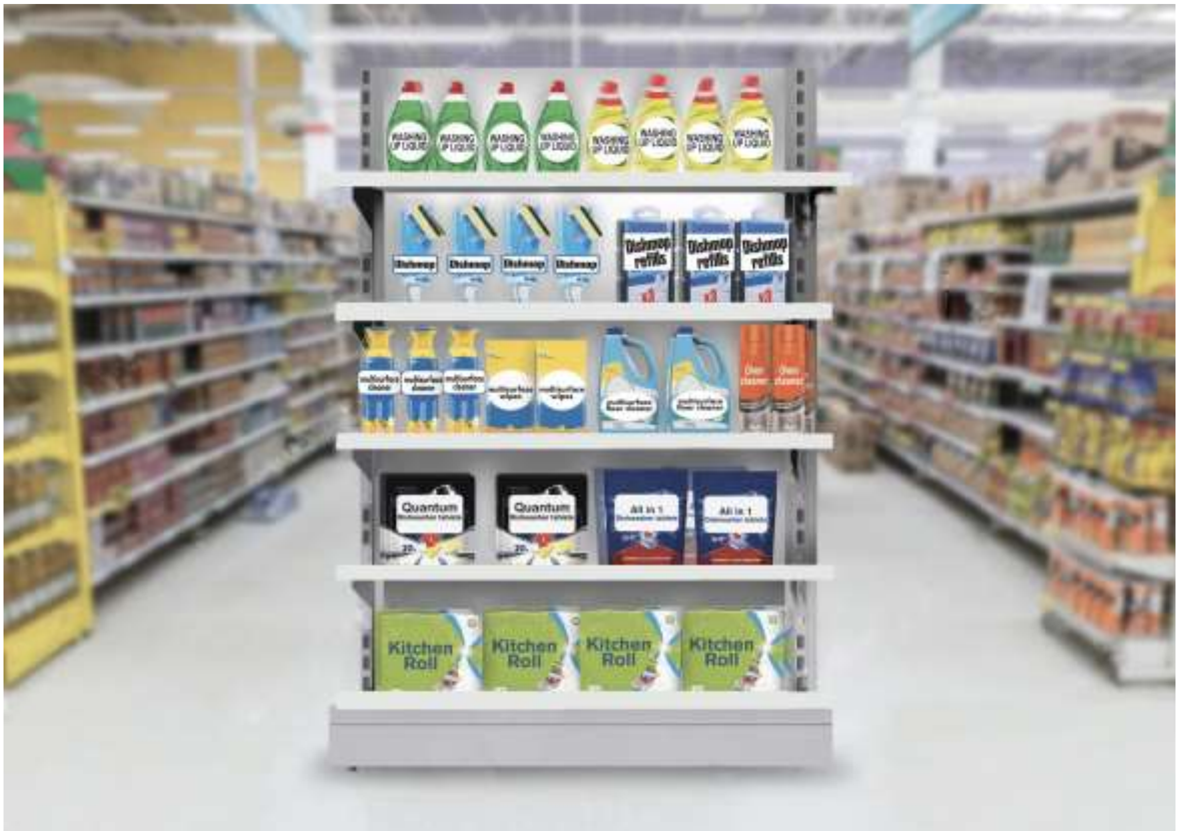

Page Break

Timer4 Timing
First Click (1)
Last Click (2)
Page Submit (3)
Click Count (4)

---

blurred_aisle_2

Page Break

Timer5 Timing
First Click (1)
Last Click (2)
Page Submit (3)
Click Count (4)

EOA1_nonfood_2

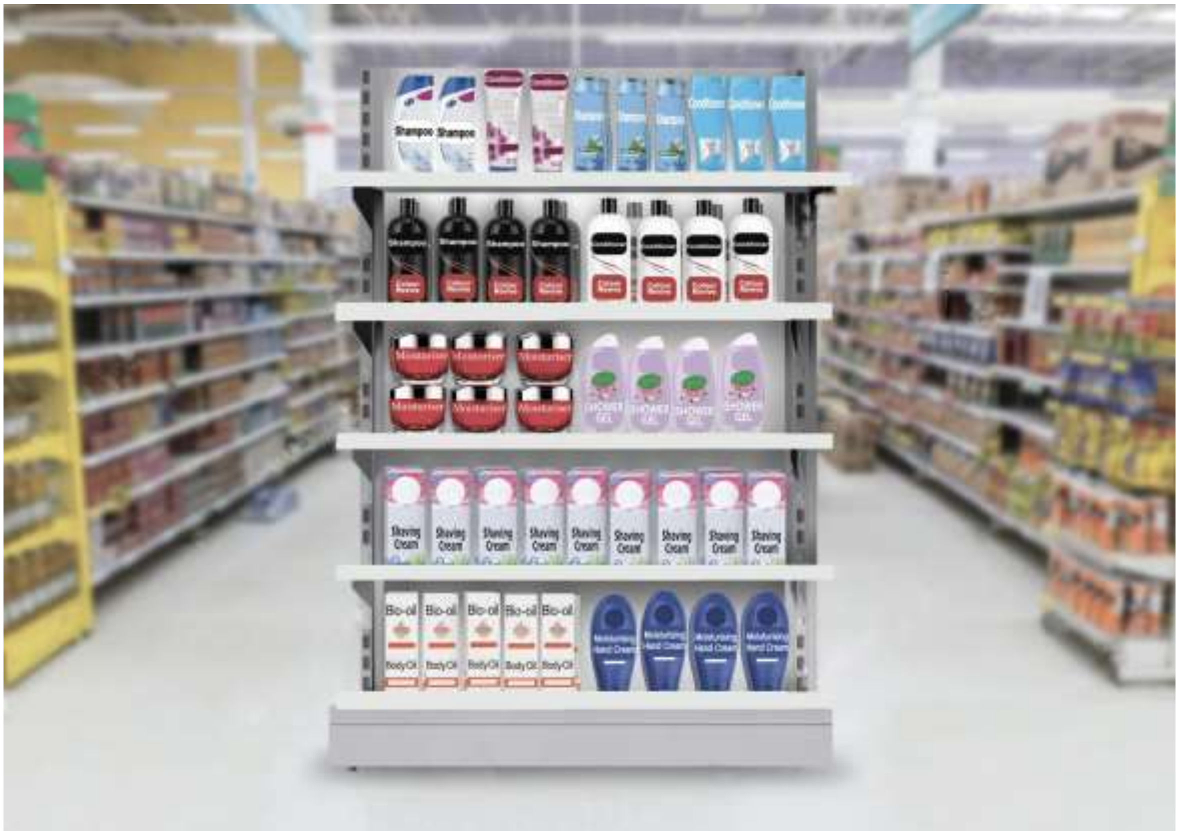

Page Break

Timer6 Timing
First Click (1)
Last Click (2)
Page Submit (3)
Click Count (4)

blurred_aisle_3

Page Break

Timer7 Timing
First Click (1)
Last Click (2)
Page Submit (3)
Click Count (4)

EOA1_nonfood_3

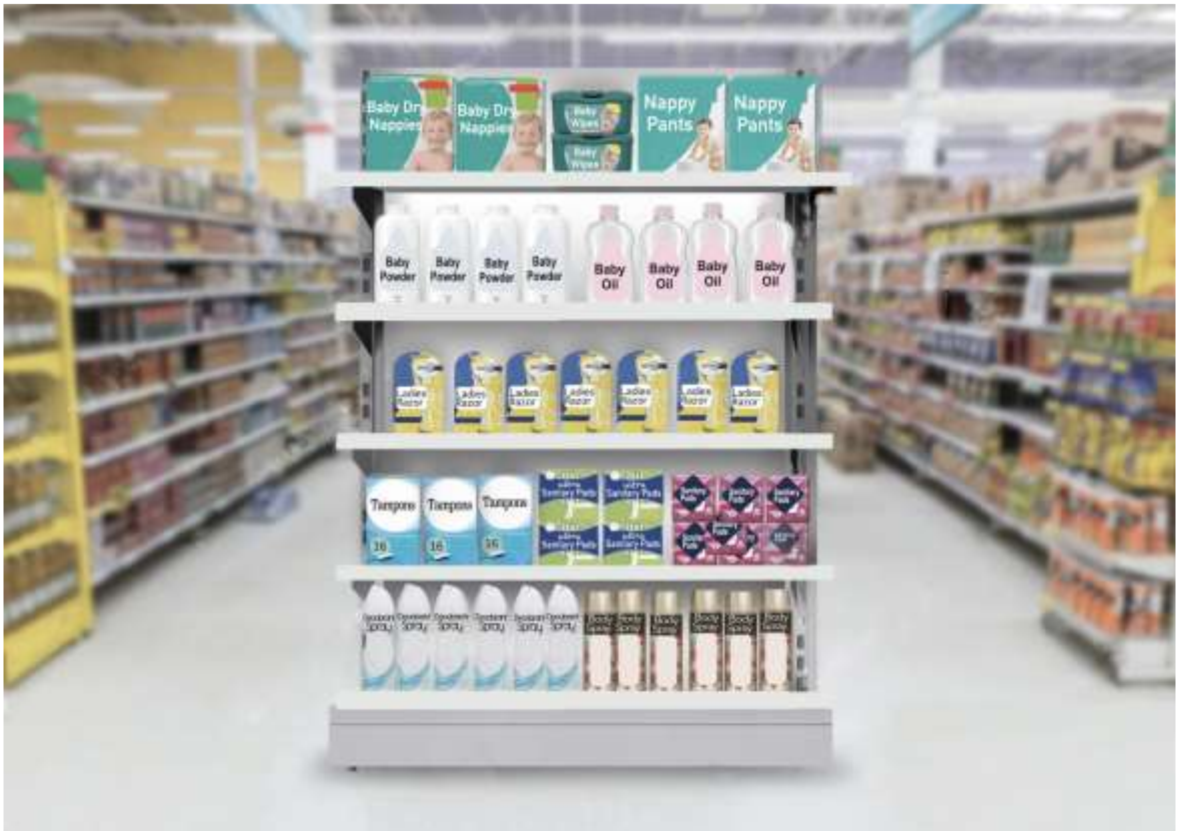

Page Break

Timer8 Timing
First Click (1)
Last Click (2)
Page Submit (3)
Click Count (4)

blurred_aisle_4

Page Break

Timer9 Timing
First Click (1)
Last Click (2)
Page Submit (3)
Click Count (4)

Checkout_nonfood1

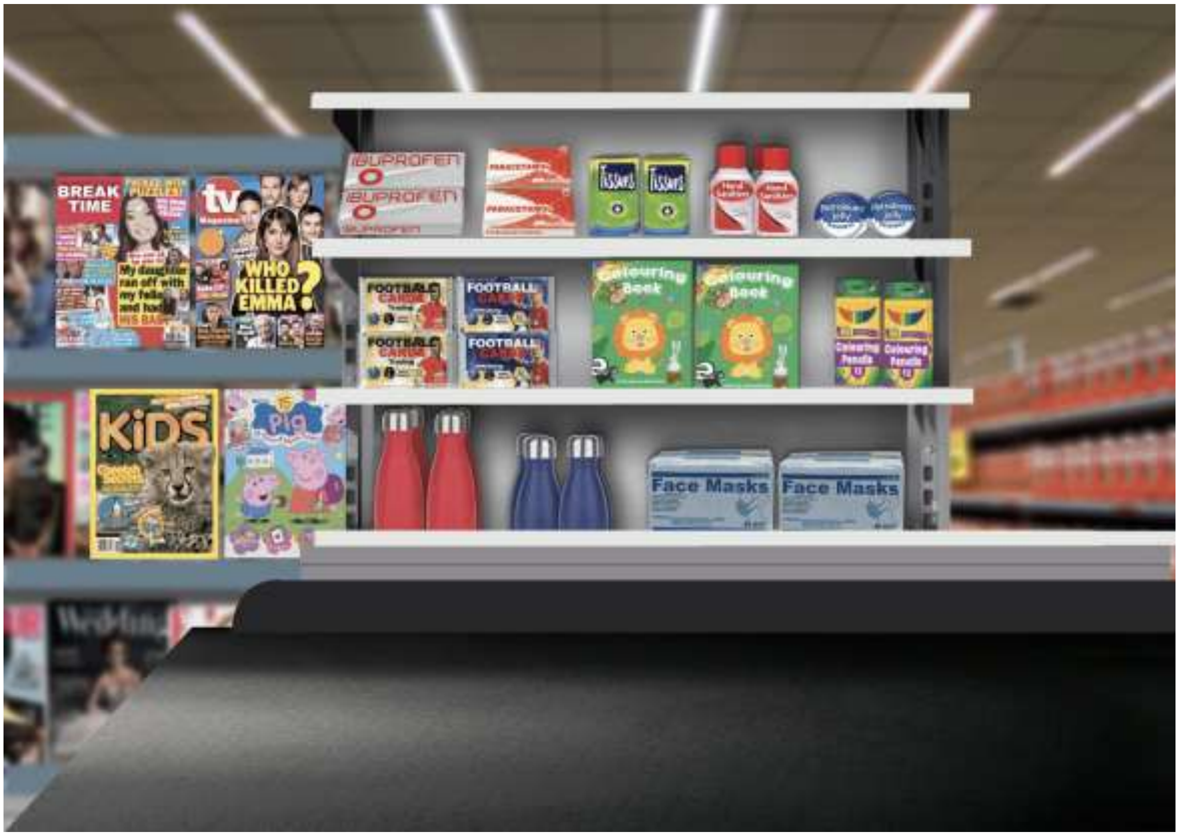

End of Block: NON FOOD1

Start of Block: End of journey 3

timer1 Timing
First Click (1)
Last Click (2)
Page Submit (3)
Click Count (4)

---

endofjourney3
You have completed store journey 3 of 6.   Thank you :)

calm_image3

End of Block: End of journey 3

Start of Block: PHASE 2 INSTRUCTIONS

timer1 Timing
First Click (1)
Last Click (2)
Page Submit (3)
Click Count (4)

phase1_end
You have completed phase 1 of the experiment.   There will now be a short break before the final phase. Please take this time to read the instructions below. **You will be doing a different task this time.**   Thank you :)

Phase2_instructions

Now you will start the second phase of the experiment.

For this phase, please click on any **products that you would purchase if this was a shopping trip.** When you have clicked, a blue dot will appear. You can select **UP TO 10** products. If you wish to remove a blue dot, you can click on it and it will disappear. If you click on more than 10, the earlier ones will start to disappear.

Each image will be displayed for 10 seconds.

In between each of these images will be a filler image of a supermarket aisle. You do not have to do anything for these images and they will only be displayed for a short amount of time (2 seconds).

End of Block: PHASE 2 INSTRUCTIONS

Start of Block: UNHEALTHY2

2Timer1 Timing
First Click (1)
Last Click (2)
Page Submit (3)
Click Count (4)

Q199

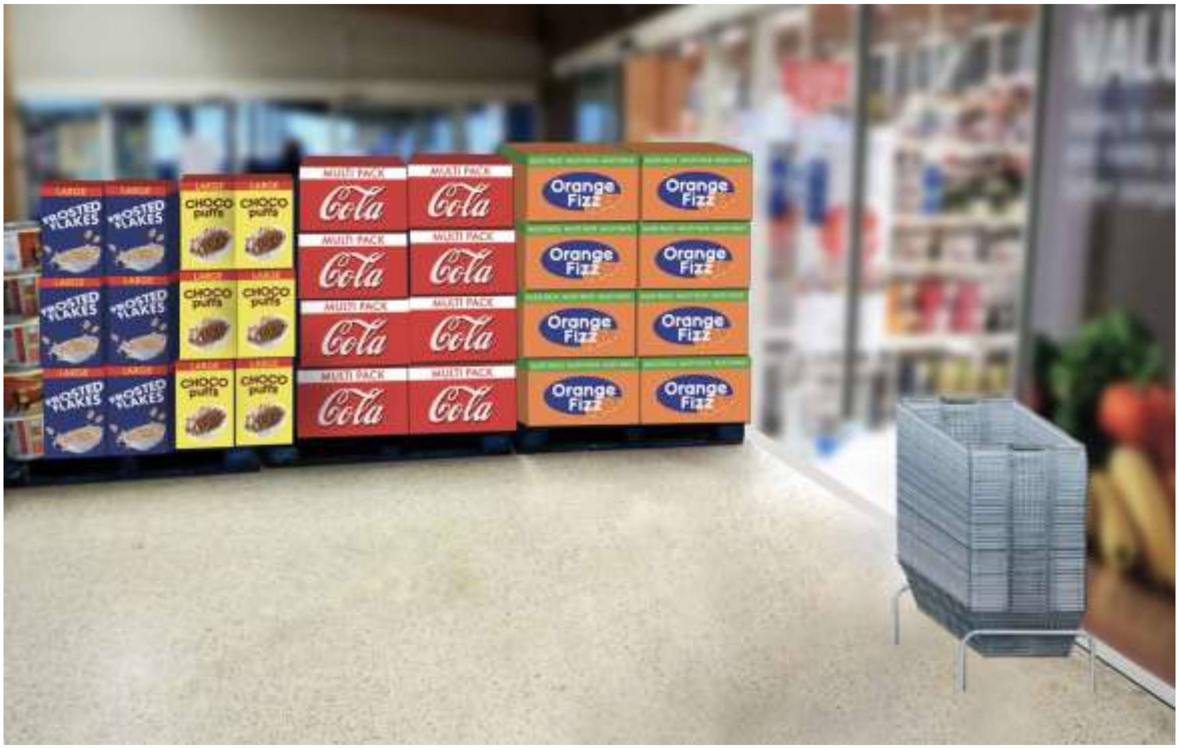

Page Break

Q200 Timing
First Click (1)
Last Click (2)
Page Submit (3)
Click Count (4)

Q201

Page Break

Q202 Timing
First Click (1)
Last Click (2)
Page Submit (3)
Click Count (4)

Q203

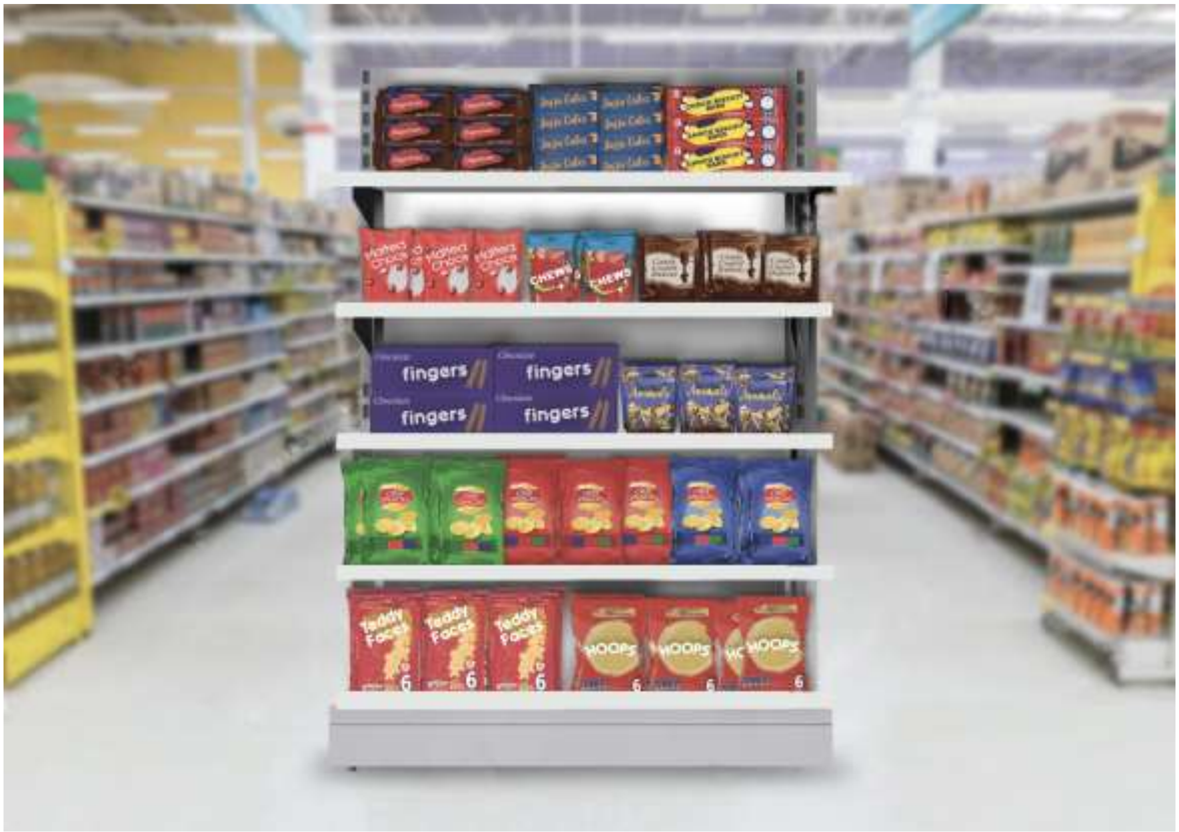

Page Break

Q204 Timing
First Click (1)
Last Click (2)
Page Submit (3)
Click Count (4)

Q205

Page Break

Q206 Timing
First Click (1)
Last Click (2)
Page Submit (3)
Click Count (4)

Q207

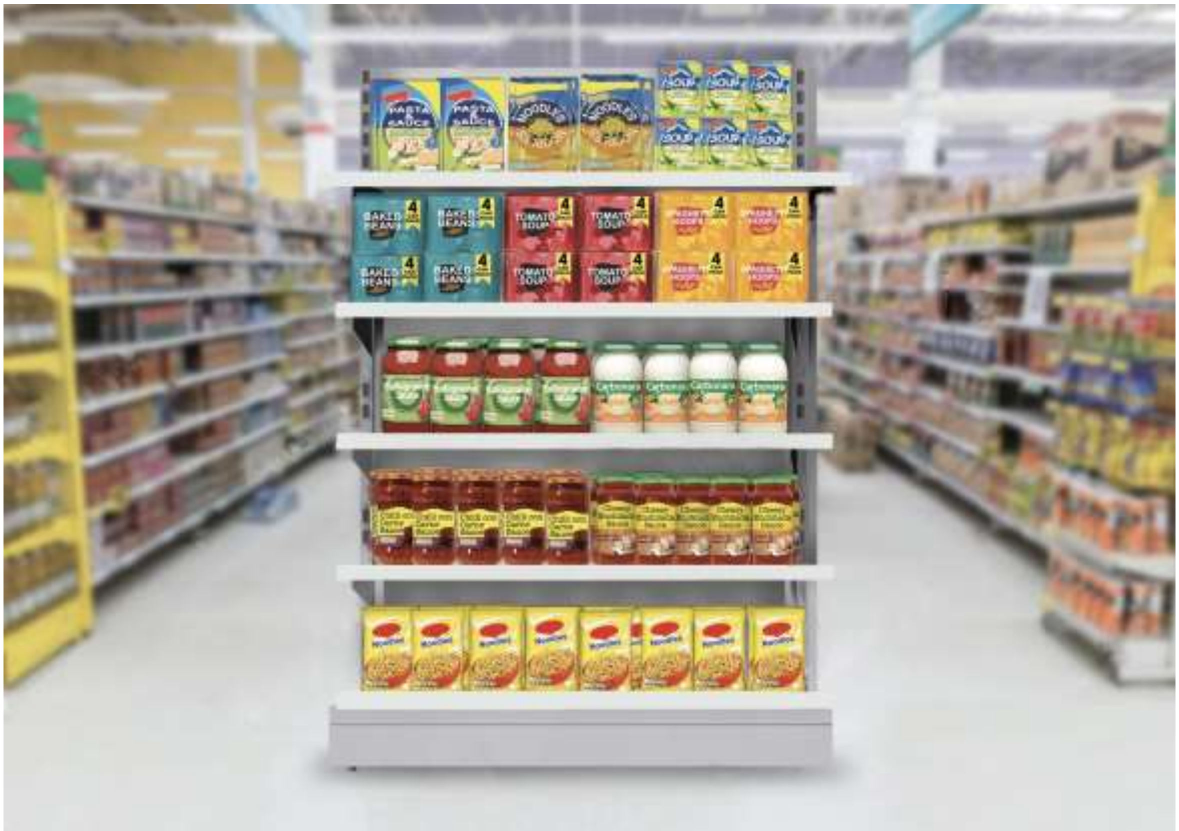

Page Break

Q208 Timing
First Click (1)
Last Click (2)
Page Submit (3)
Click Count (4)

Q209

Page Break

Q210 Timing
First Click (1)
Last Click (2)
Page Submit (3)
Click Count (4)

Q211

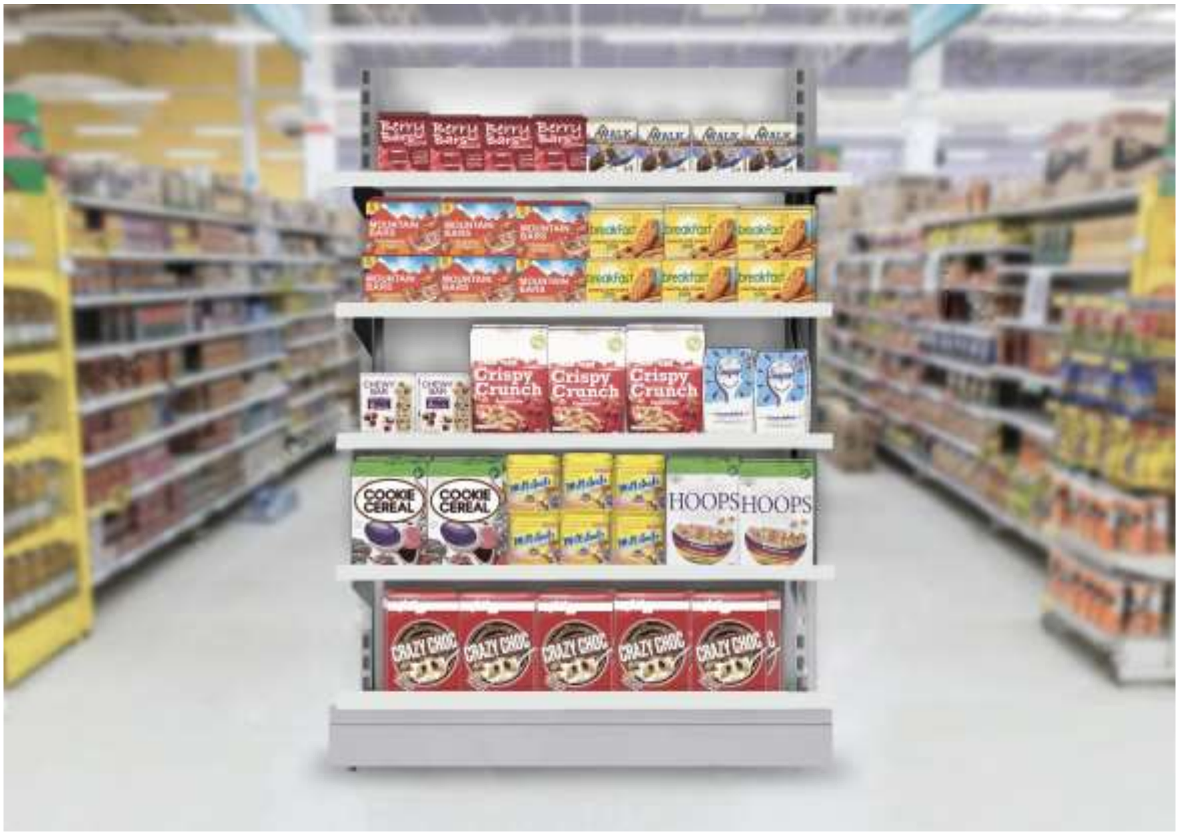

Page Break

Q212 Timing
First Click (1)
Last Click (2)
Page Submit (3)
Click Count (4)

Q213

Page Break

Q214 Timing
First Click (1)
Last Click (2)
Page Submit (3)
Click Count (4)

Q215

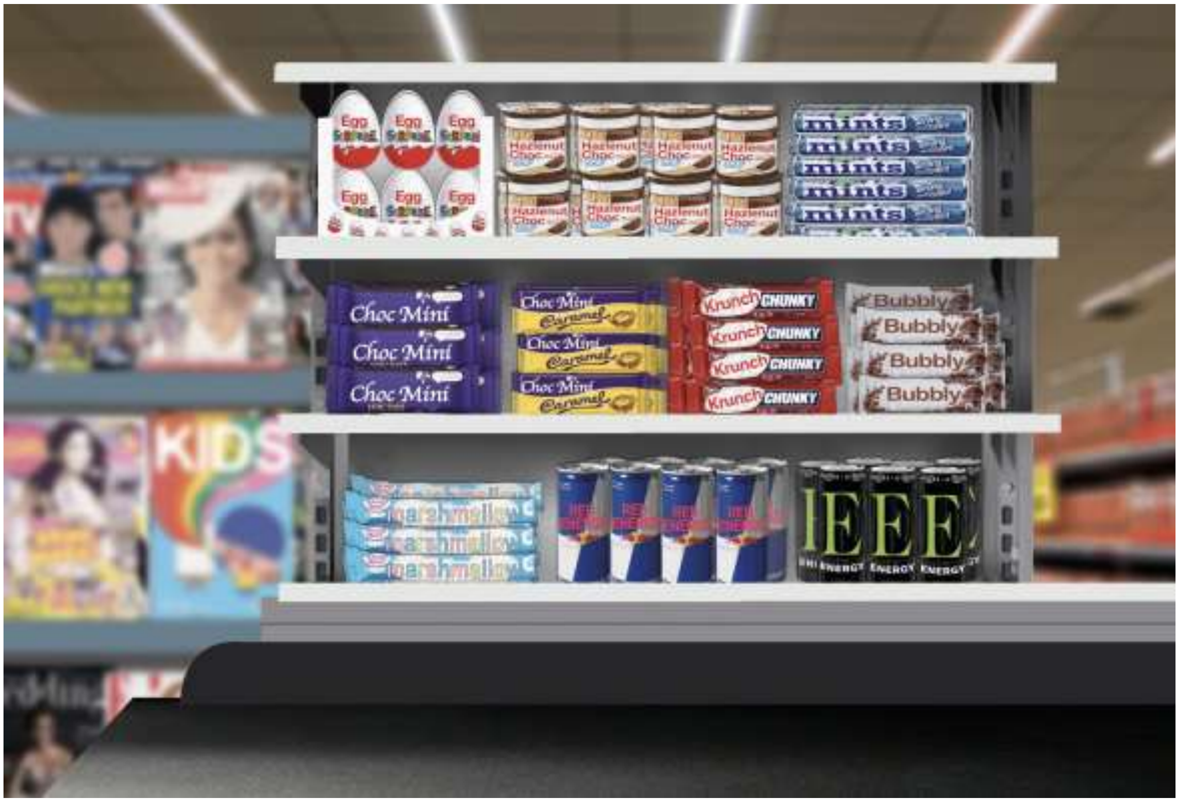

End of Block: UNHEALTHY2

Start of Block: End of journey 4

timer1 Timing
First Click (1)
Last Click (2)
Page Submit (3)
Click Count (4)

Endofjourney4
You have completed store journey 4 of 6. There will now be a 20 second break before the next journey. Thank you :)

calm_image4

End of Block: End of journey 4

Start of Block: HEALTHY2

Q216 Timing
First Click (1)
Last Click (2)
Page Submit (3)
Click Count (4)

Q217

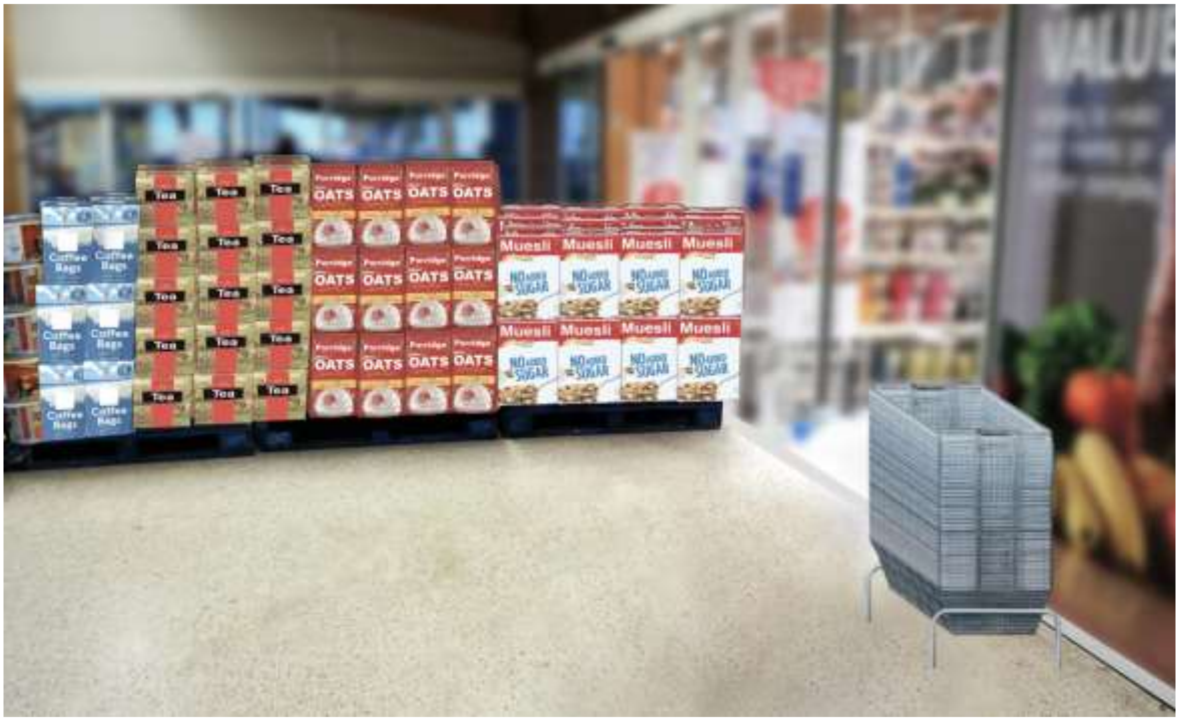

Page Break

Q218 Timing
First Click (1)
Last Click (2)
Page Submit (3)
Click Count (4)

Q219

Page Break

Q220 Timing
First Click (1)
Last Click (2)
Page Submit (3)
Click Count (4)

Q221

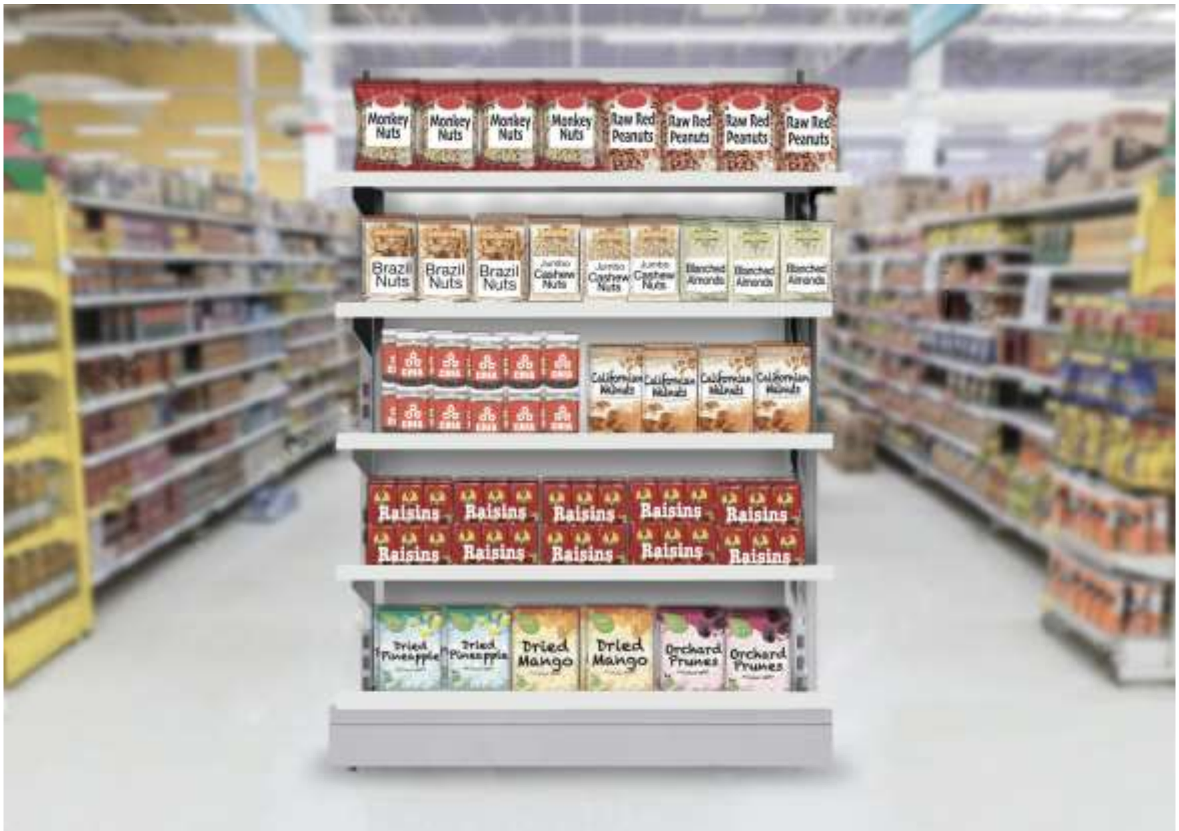

Page Break

Q222 Timing
First Click (1)
Last Click (2)
Page Submit (3)
Click Count (4)

Q223

Page Break

Q224 Timing
First Click (1)
Last Click (2)
Page Submit (3)
Click Count (4)

Q225

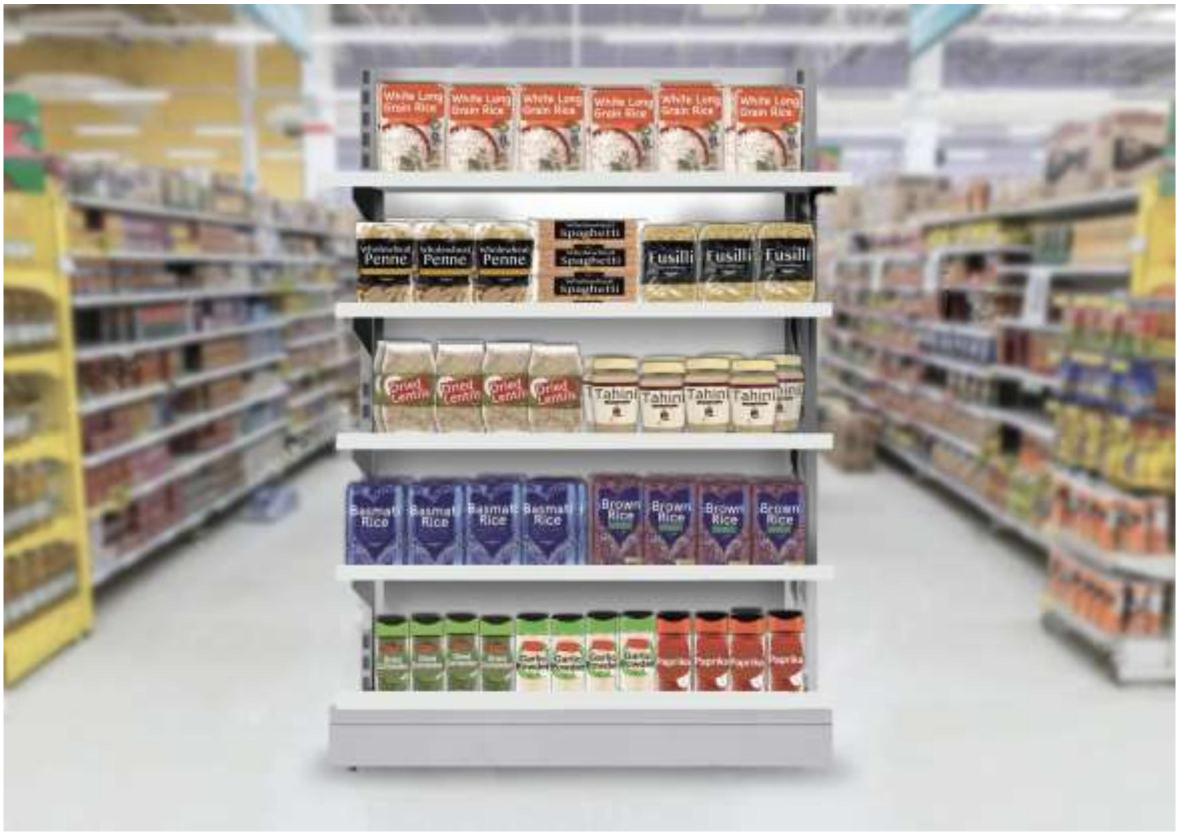

Page Break

Q226 Timing
First Click (1)
Last Click (2)
Page Submit (3)
Click Count (4)

Q227

Page Break

Q228 Timing
First Click (1)
Last Click (2)
Page Submit (3)
Click Count (4)

Q229
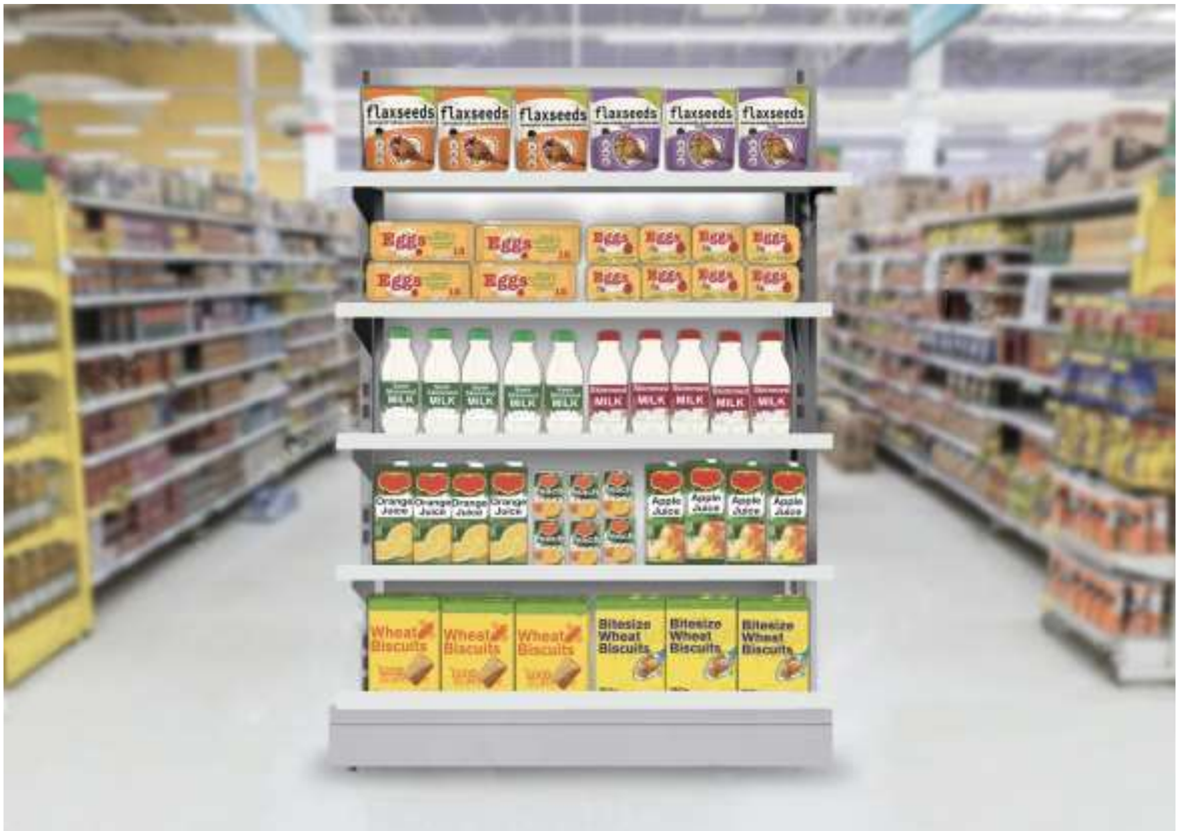

Page Break

Q230 Timing
First Click (1)
Last Click (2)
Page Submit (3)
Click Count (4)

Q231

Page Break

Q232 Timing
First Click (1)
Last Click (2)
Page Submit (3)
Click Count (4)

Q233

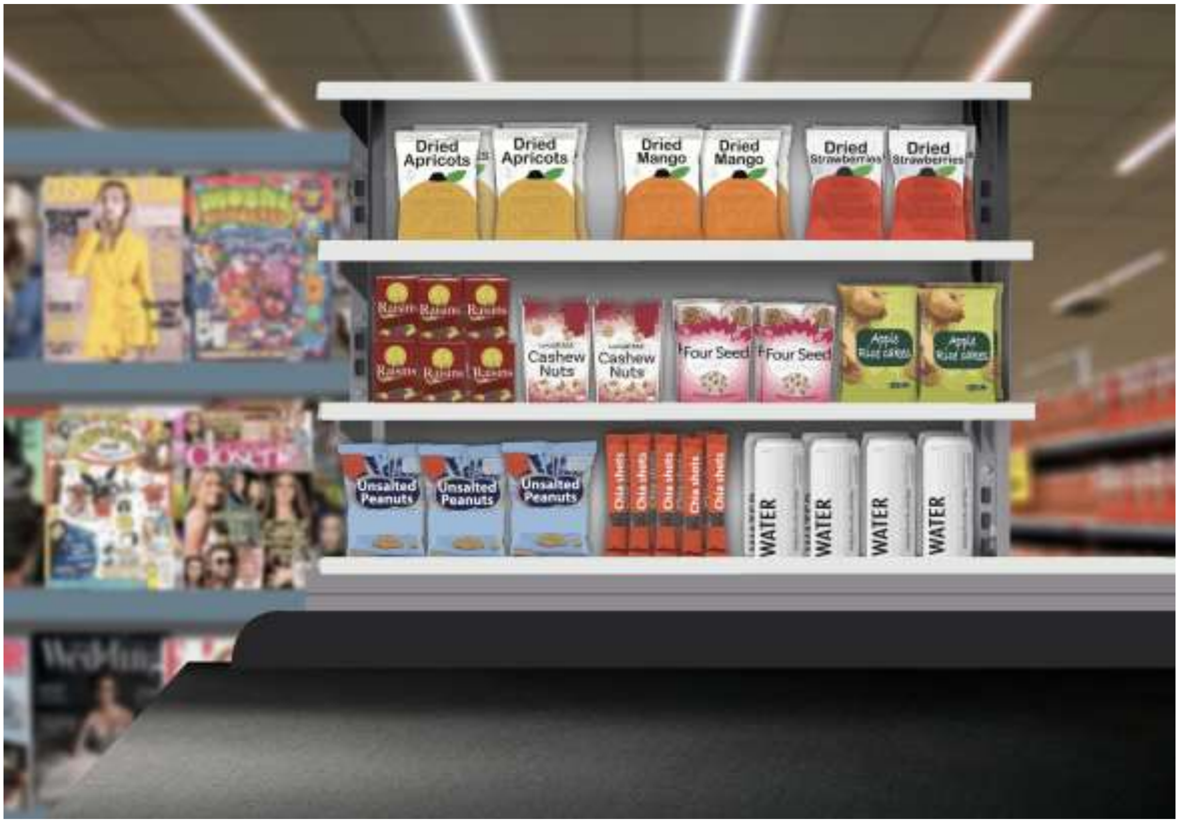

End of Block: HEALTHY2

Start of Block: End of journey 5

Timer1 Timing
First Click (1)
Last Click (2)
Page Submit (3)
Click Count (4)

---

endofjourney5
You have completed store journey 5 of 6. There will now be a 20 second break before the next journey. Thank you :)

calm_image5

End of Block: End of journey 5

Start of Block: NONFOOD2

Q234 Timing
First Click (1)
Last Click (2)
Page Submit (3)
Click Count (4)

Q235
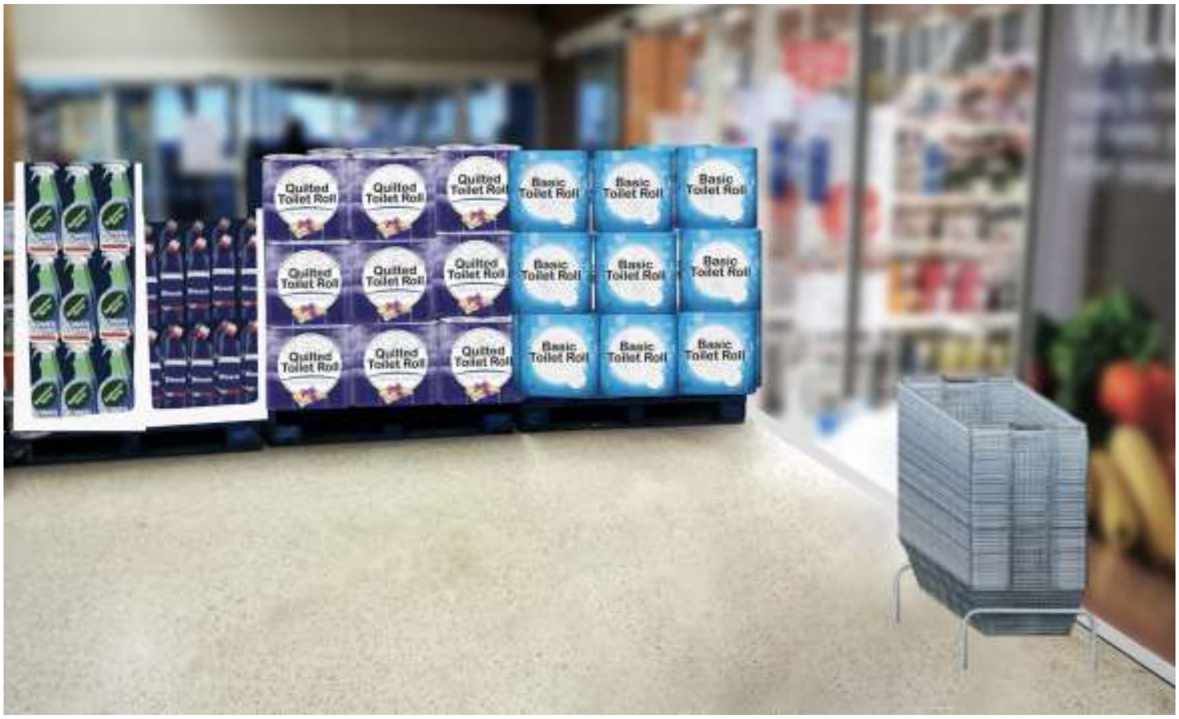

Page Break

Q236 Timing
First Click (1)
Last Click (2)
Page Submit (3)
Click Count (4)

Q237

Page Break

Q238 Timing
First Click (1)
Last Click (2)
Page Submit (3)
Click Count (4)

Q239

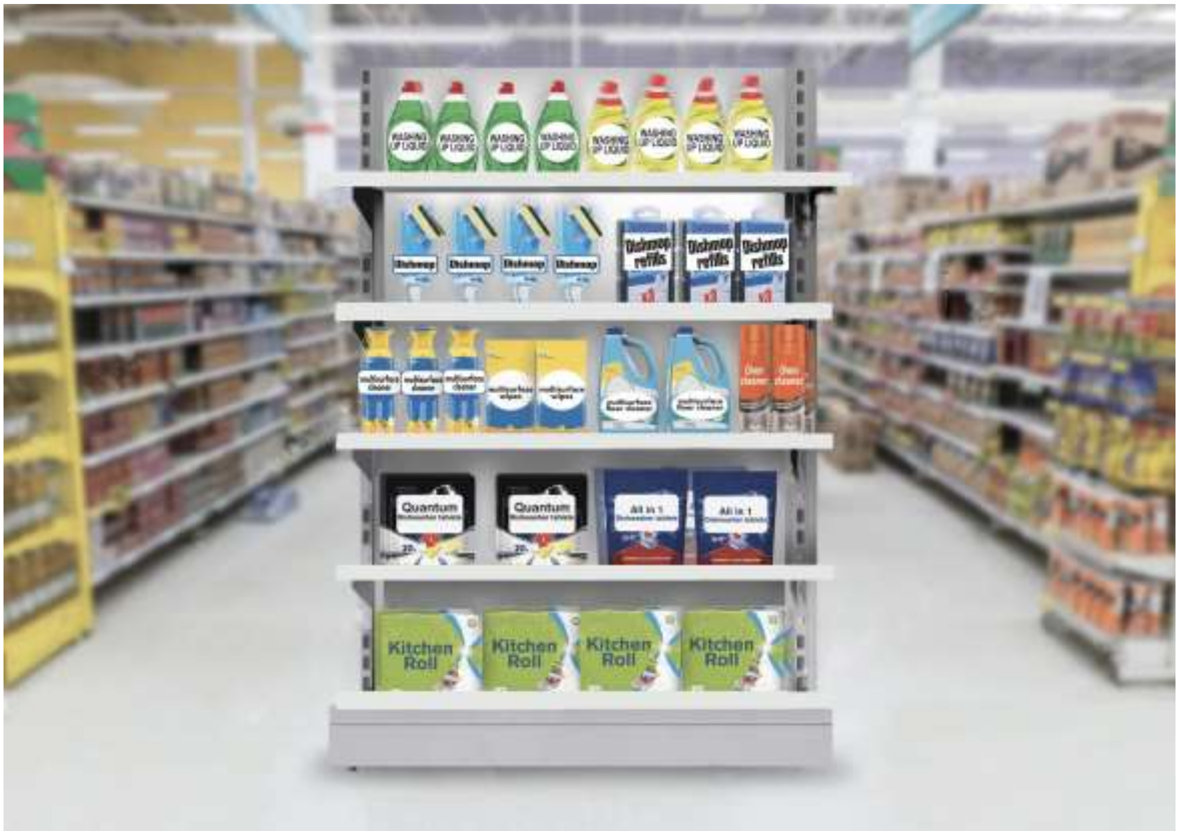

Page Break

Q240 Timing
First Click (1)
Last Click (2)
Page Submit (3)
Click Count (4)

Q241

Page Break

Q242 Timing
First Click (1)
Last Click (2)
Page Submit (3)
Click Count (4)

Q243

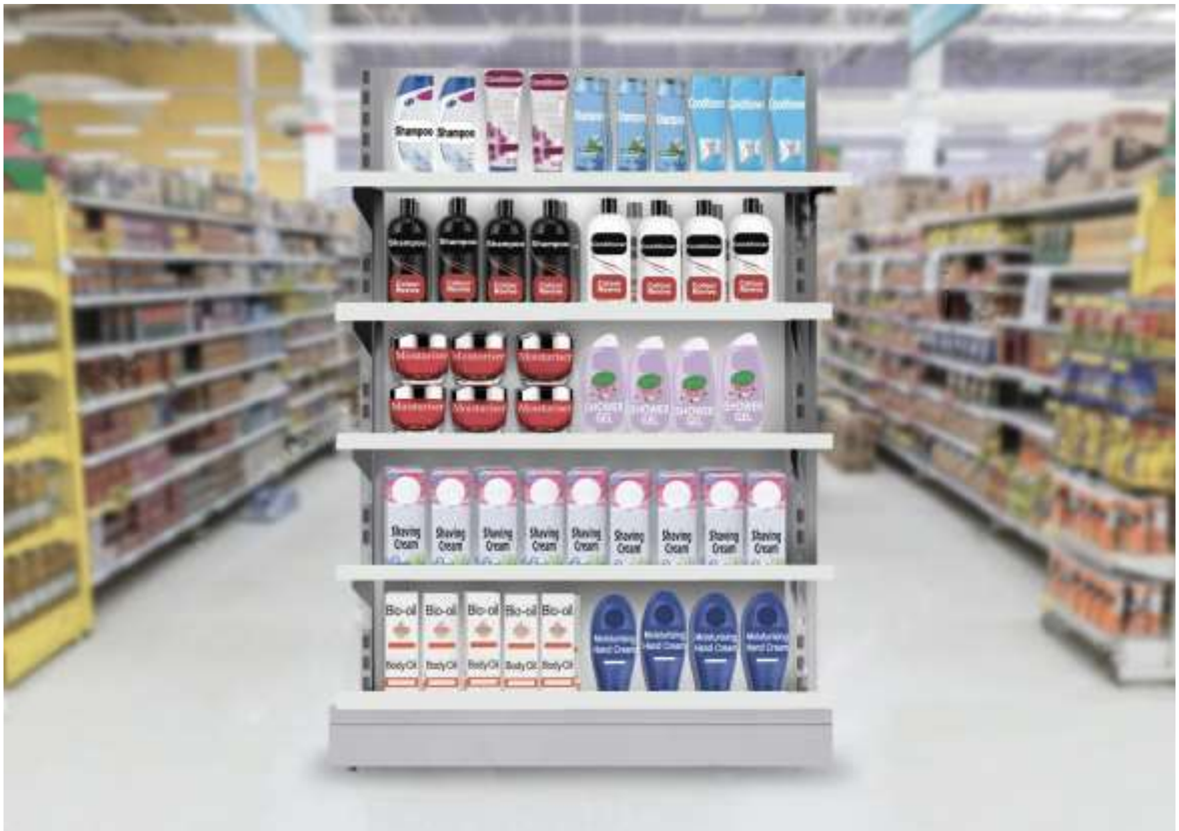

Page Break

Q244 Timing
First Click (1)
Last Click (2)
Page Submit (3)
Click Count (4)

Q245

Page Break

Q246 Timing
First Click (1)
Last Click (2)
Page Submit (3)
Click Count (4)

Q247

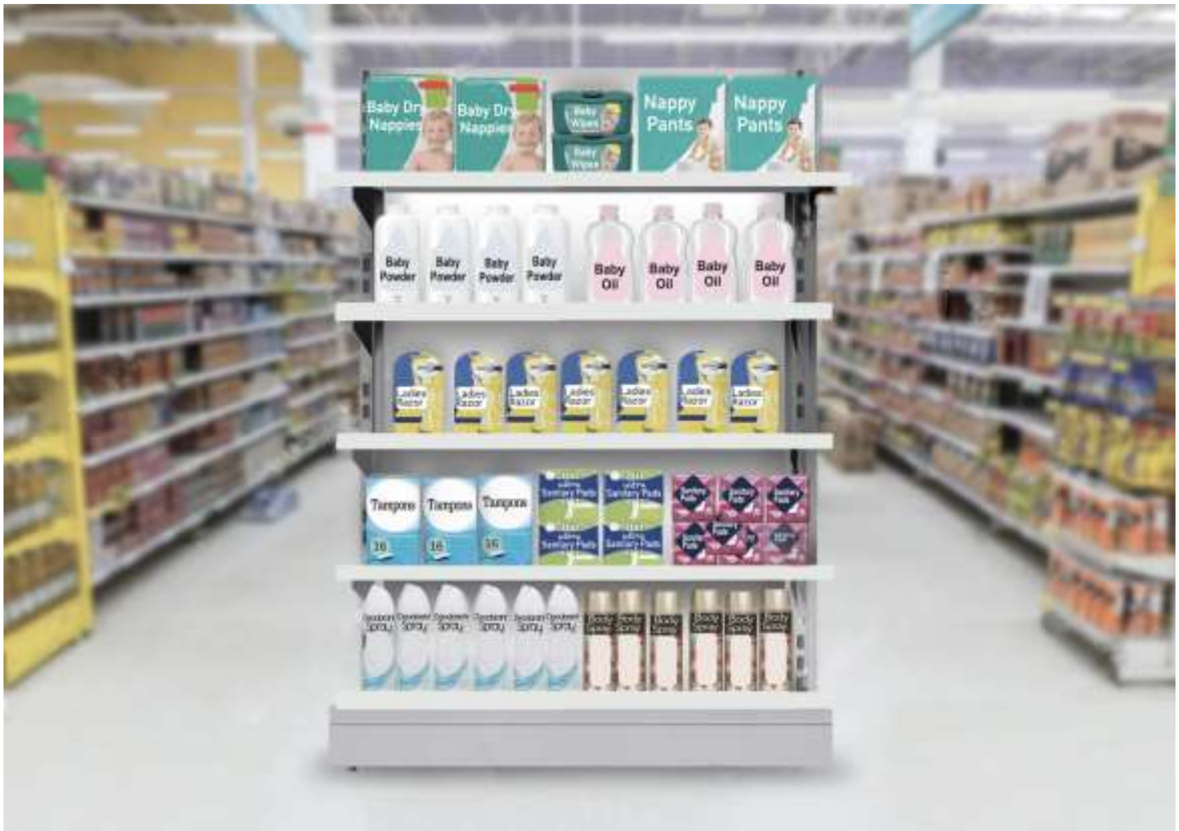

Page Break

Q248 Timing
First Click (1)
Last Click (2)
Page Submit (3)
Click Count (4)

Q249

Page Break

Q250 Timing
First Click (1)
Last Click (2)
Page Submit (3)
Click Count (4)

Q251
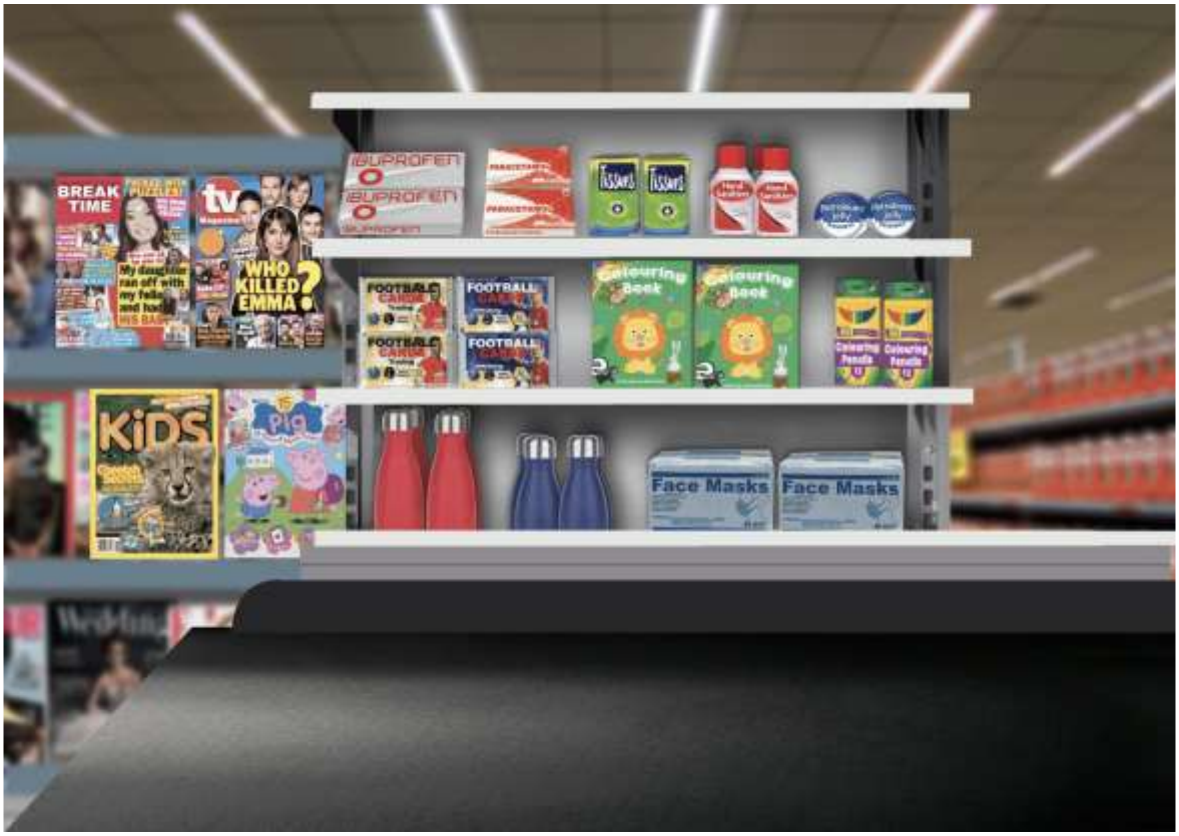

End of Block: NONFOOD2

Start of Block: End of journey 6

timer1 Timing
First Click (1)
Last Click (2)
Page Submit (3)
Click Count (4)

---

endofjourney6
You have completed store journey 6 of 6.   Thank you :)

Calm_image6

End of Block: End of journey 6
