# Supplementary material for: Women intend to buy, and attend more to, healthy foods in supermarkets: attention to product placement observed in women in a 2-dimensional simulated supermarket environment
Source: BMC Public Health. 2026 May 18;26:2131. doi: 10.1186/s12889-026-27679-5 (PMC13362013; doi:10.1186/s12889-026-27679-5)
Supplement: Supplementary file 3 — Supplementary Material 3. [file 12889_2026_27679_MOESM3_ESM.pdf]

APPROVE Phase 2 – post-study questionnaire

About you

In this section, we want to learn a little bit about you.

1.1 What is your date of birth (DD/MMYY)?

☐☐☐☐☐☐

1.2 To which of the following ethnic groups do you feel you belong?

White British	☐	Bangladeshi	☐
White other *(please specify)* ___________	☐	Pakistani	☐
		Chinese	☐
Black Caribbean	☐	Other Asian group *(please specify)* ___________	☐
Black African	☐		
Black other *(please specify)* ___________	☐	Mixed	☐
		Indian	☐
Arabic	☐	Other, (*please specify)* ___________	☐

1.3 Have you passed any exams or formal qualifications? ☐
[only place number which corresponds to highest qualification in the box]

1. None
2. GCSE grade D or lower/ NVQ1/ Foundation GNVQ/Scottish standard grade foundation/ School cert
3. GCSE grade A,B,C/ RSA secretarial/ NVQ2/Intermediate GNVQ/Scottish standard grade general or credit/ Matric
4. A levels/ AS Level/ City & Guilds/ EN(G)/ ONC/ NNEB/ BTech (day release)/ NVQ3/ Advanced GNVQ/ OND / HNC
5. HND/ RGN/ NVQ4
6. Degree/ NVQ5
7. PGCE/Postgraduate degree (e.g. Masters, PhD etc.)
8. Other (specify)______________________

1.4 Which of the following best describes your present work situation?

- Employed ☐
- Self-employed ☐
- Self-employed off sick ☐
- Employed off sick (paid) ☐
- Employed off sick (unpaid) ☐
- Maternity leave ☐
- Unemployed ☐
- Other unpaid (please specify below) ☐

____

1.5 How many people live in your household?

1.5.1 Number of adults ☐

1.5.2 Number of children (please include any children that may only live with you part-time e.g. your partner's children at weekends)

☐

1.5.3 What are the dates of birth for each child in your household (DD/MM/YY)?

1. ☐☐ ☐☐ ☐☐
2. ☐☐ ☐☐ ☐☐
3. ☐☐ ☐☐ ☐☐
4. ☐☐ ☐☐ ☐☐
5. ☐☐ ☐☐ ☐☐

1.6 Do you or your partner receive Universal Credit?

0. No ☐

1. Yes ☐

1.7 What is your home postcode? ____________________

Your eating habits

In this section we would like to know about some of the foods you eat and how often you eat them.
[If more than once a day option is selected, ask the total number of times per day consumed and write in the box provided.]

2.1 OVER THE PAST MONTH HOW OFTEN HAVE YOU EATEN THESE FOODS?

	Over the past month	Never	Once a Month	Once every two weeks	1-2 Times per Week	3-6 Times per Week	Once a day	More than once a day
2.1.1	Peppers and watercress							☐
2.1.2	Tomatoes							☐
2.1.3	Vegetable dishes							☐
2.1.4	Courgettes, marrows and leeks							☐
2.1.5	Green salad							☐
2.1.6	Brown and wholemeal bread							☐
2.2.7	Average number of slices per serving							Slices
2.1.8	Onions							☐
2.1.9	Vegetarian foods							☐
2.1.10	Pasta and dumplings							☐
2.1.11	Spinach							☐
2.1.12	Full-fat liquid milk							☐
2.1.13	How much **full-fat milk** on average do you use per day in your drinks, added to breakfast cereals, etc?							pints
2.1.14	Crisps and savoury snacks							☐
2.1.15	Yorkshire puddings and savoury pancakes							☐
2.1.16	White bread							☐
2.1.17	Average number of slices per serving							

2.1.18	Sugar							☐
2.1.17	How many teaspoons of sugar do you add each day to breakfast cereals, tea, coffee, etc.?							teaspoons
	**Over the past month**	**Never**	**Once a Month**	**Once every two weeks**	**1-2 Times per Week**	**3-6 Times per Week**	**Once a day**	**More than once a day**
2.1.18	Gravy granules and powders							☐
2.1.19	Sausages							☐
2.1.20	Meat pies							☐
2.1.21	Beef							☐
2.1.22	Chips and roast potatoes							☐
2.1.23	How many portions of chips per serving (Regular McDonalds size)?							☐
2.1.24	How many egg sized potatoes per serving							☐
2.1.25	Cakes, pies, puddings or pastries							☐
2.1.26	Sweets or chocolate							☐
2.1.27	Fizzy drinks, or soft drinks like squash *[doesn't include diet or sugar-free drinks]*							☐

We would like to know how many portions of fruit and vegetables that you eat on a typical day.

2.2.1 How many pieces of fruit, of any sort, do you eat on a typical day? ☐☐
[A portion constitutes 80 g or 1 apple, banana, pear or similar sized fruit or 30 g of dried fruit. Also includes one small 150ml glass of fruit juice or smoothie.]

2.2.2 How many portions of vegetables, excluding potatoes, do you eat on a typical day? ☐☐
[A portion constitutes either 3 heaped table spoons of vegetables or a desert bowl of salad.]

Shopping

In this section we want to understand a little about your food shopping habits.

We would like to know what products you buy from the checkout at the supermarket.

3.1 Please select the option that is most true to you:

		Always	Sometimes	Never
**3.1.1**	Do you browse products displayed at checkouts?			
**3.1.2**	Do you buy sugary drinks when displayed at checkouts?			
**3.1.3**	Do you buy confectionery when displayed at checkouts?			
**3.1.4**	Do you buy water or sugar free drinks when displayed at checkouts?			
**3.1.5**	Do you buy fruit when displayed at checkouts?			
**3.1.6**	Do you buy non-food items when displayed at checkouts?			

3.2 What are your reasons for browsing products at checkouts?

To keep occupied ☐ Didn't intend to buy but fancied it ☐

Forgot to buy in aisle ☐ Do not browse ☐

Other (please specify in the box below):
