# Supplementary material for: Women intend to buy, and attend more to, healthy foods in supermarkets: attention to product placement observed in women in a 2-dimensional simulated supermarket environment
Source: BMC Public Health. 2026 May 18;26:2131. doi: 10.1186/s12889-026-27679-5 (PMC13362013; doi:10.1186/s12889-026-27679-5)
Supplement: Supplementary file 4 — Supplementary Material 4. [file 12889_2026_27679_MOESM4_ESM.docx]

**Possible supplementary table: Median (IQR) clicks (n = 201)**

|  | **Healthy** | ***Unhealthy*** | ***Non-food*** |
| --- | --- | --- | --- |
| ***Products woman is interested in (attention), number of clicks per 10 items*** |  |  |  |
| **Entrance** | 2.5 (2.5, 5.0) | 5.0 (2.5, 7.5) | 5.0 (2.5, 5.0) |
| **End-of-aisle** | 2.1 (1.2, 3.0) | 2.3 (1.4, 3.1) | 2.1 (1.2, 3.3) |
| **Checkout** | 2.0 (1.0, 4.0) | 3.0 (1.0, 4.0) | 1.9 (1.3, 2.5) |
| **Total** | 2.1 (1.5, 3.4) | 2.4 (1.6, 3.5) | 2.1 (1.3, 3.0) |
| ***Products woman would like to buy (intention), number of clicks per 10 items*** |  |  |  |
| **Entrance** | 5.0 (2.5, 5.0) | 2.5 (0.0, 5.0) | 5.0 (2.5, 7.5) |
| **End-of-aisle** | 2.4 (1.8, 3.6) | 2.0 (1.4, 3.1) | 2.1 (1.5, 3.0) |
| **Checkout** | 2.0 (1.0, 3.0) | 2.0 (1.0, 3.0) | 1.3 (0.6, 2.5) |
| **Total** | 2.6 (1.7, 3.4) | 2.2 (1.4, 3.1) | 2.3 (1.5, 3.0) |

**Another possible supplementary table: Median (IQR) clicks (n = 71)**

|  | **Healthy** | ***Unhealthy*** | ***Non-food*** |
| --- | --- | --- | --- |
| ***Viewing time (attention) (seconds)*** |  |  |  |
| **Entrance** | 8.3 (7.6, 8.6) | 8.2 (7.8, 8.5) | 8.2 (7.7, 8.7) |
| **End-of-aisle** | 24.9 (24.0, 25.5) | 24.8 (23.6, 25.4) | 24.8 (23.9, 25.5) |
| **Checkout** | 8.3 (7.9, 8.6) | 8.2 (7.9, 8.5) | 8.3 (7.9, 8.6) |
| **Total** | 41.3 (39.5, 42.4) | 41.0 (39.3, 42.1) | 41.0 (39.6, 42.2) |
| ***Products woman would like to buy (intention), number of clicks per 10 items*** |  |  |  |
| **Entrance** | 2.5 (2.5, 5.0) | 2.5 (0.0, 2.5) | 2.5 (0.0, 5.0) |
| **End-of-aisle** | 1.8 (1.2, 2.4) | 1.1 (0.6, 1.7) | 0.9 (0.6, 1.5) |
| **Checkout** | 1.0 (0.0, 3.0) | 1.0 (0.0, 2.0) | 1.3 (0.6, 1.9) |
| **Total** | 1.9 (1.3, 2.6) | 1.0 (0.6, 1.8) | 1.3 (0.8, 1.7) |
